# Mine water inrush source discrimination model based on KPCA-ISSA-KELM

**DOI:** 10.1371/journal.pone.0299476

**Published:** 2024-06-03

**Authors:** Wei Wang, Xinchao Cui, Yun Qi, Kailong Xue, Ran Liang, Zhipeng Sun, Hongjing Tao

**Affiliations:** 1 College of Mechanical Engineering and Automation, Liaoning University of Technology, Jinzhou, P.R. China; 2 School of Coal Engineering, Shanxi Datong University, Datong, P.R. China; 3 China Safety Science Journal Editorial Department, China Occupational Safety and Health Association, Beijing, P.R. China; UNITEN: Universiti Tenaga Nasional, MALAYSIA

## Abstract

In order to ensure the safety of coal mine production, a mine water source identification model is proposed to improve the accuracy of mine water inrush source identification and effectively prevent water inrush accidents based on kernel principal component analysis (KPCA) and improved sparrow search algorithm (ISSA) optimized kernel extreme learning machine (KELM). Taking Zhaogezhuang mine as the research object, firstly, Na^+^, Ca^2+^, Mg^2+^, Cl^-^, SO2- 4 and HCO- 3 were selected as evaluation indexes, and their correlation was analyzed by SPSS27 software, with reducing the dimension of the original data by KPCA. Secondly, the Sine Chaotic Mapping, dynamic adaptive weights, and Cauchy Variation and Reverse Learning were introduced to improve the Sparrow Search Algorithm (SSA) to strengthen global search ability and stability. Meanwhile, the ISSA was used to optimize the kernel parameters and regularization coefficients in the KELM to establish a mine water inrush source discrimination model based on the KPCA-ISSA-KELM. Then, the mine water source data are input into the model for discrimination in compared with discrimination results of KPCA-SSA-KELM, KPCA-KELM, ISSA-KELM, SSA-KELM and KELM models. The results of the study show as follows: The discrimination results of the KPCA-ISSA-KELM model are in agreement with the actual results. Compared with the other models, the accuracy of the KPCA-ISSA-KELM model is improved by 8.33%, 12.5%, 4.17%, 21.83%, and 25%, respectively. Finally, when these models were applied to discriminate water sources in a coal mine in Shanxi, and the misjudgment rates of each model were 28.57%, 19.05%, 14.29%, 23.81%, 9.52% and 4.76%, respectively. From this, the KPCA-ISSA-KLEM model is the most accurate about discrimination and significantly better than other models in other evaluation indicators, verifying the universality and stability of the model. It can be effectively applied to the discrimination of inrush water sources in mines, providing important guarantees for mine safety production.

## 1 Introduction

Mine water inrush, a common disaster accident in coal mine production, is one of the main threats to coal mine safety production [1]. The complexity of the geological conditions of the mine, the instability of the hydrogeological conditions and the irrationality of the mining process and other factors have become an important reason for inducing the occurrence of mine water inrush accidents. Once a mine water inrush accident occurs, it will not only disturb the normal production order of the mine, but also may trigger serious casualties and economic losses. According to incomplete statistics, there were 95 coal mine flooding accidents nationwide with 536 deaths between 2011 and 2021 [2], resulting in economic losses of billions of yuan, after gas accidents. The discrimination of water inrush source in mines can predicts accurately the amount and duration of water inrush as soon as possible, thus taking more effective prevention and control measures to ensure the safety of mine production. At the same time, it is possible to better understand the distribution and flow of groundwater, taking more reasonable drainage measures and reduce waste and pollution of groundwater. Therefore, a scientific and effective method to accurately identify the source of inrush water plays a crucial guiding role in the management of mine water hazards [3].

At present, groundwater chemical analysis, hydrodynamic analysis, geophysical exploration, laser-induced fluorescence technology, water temperature analysis is usually used to discriminate the water-inrush source [4], of which groundwater chemical analysis is widely used because of the convenience of collecting samples and the ability of reflecting the essential characteristics of groundwater. In recent years, metaheuristic algorithms have been widely applied under the background of booming development of artificial intelligence. Lai Vivien et al. [5] used various metaheuristic algorithms to study reservoir optimization, demonstrating their potential applications and performance in reservoir operation optimization. Ibrahim Karim H et al. [6] used various artificial intelligence models to predict and analyze unstable patterns in the hydrological field, while exploring data fusion methods and hybrid artificial intelligence and optimization modeling techniques for flow prediction. At the same time, more and more scholars are beginning to integrate mathematical and statistical methods with machine learning. Based on hydrochemical characteristics, a model for inrush water sources discrimination is constructed, continuously improving the accuracy of identifying inrush water sources. XU Xing et al. [7] proposed a water-inrush source discrimination model based on ACPSO-BPNN by optimizing the weights and thresholds in BP neural network (BPNN) by Particle Swarm Algorithm (PSO). The results showed that the model is higher accuracy and generalization, which provides a powerful auxiliary decision support for promoting mine water prevention and control. GUO Zhong’an et al. [8] reduced the dimension of the aquifer data of the 51101 working face in Liangzhuang coal mine by Principal Component Analysis (PCA) and optimized the input weights and thresholds of Extreme Learning Machines by Genetic Algorithm (GA), finally constructing a water inrush source discrimination model based on PCA-GA-ELM, applying the model to the 1303 working face of Lilou Coal Mine, which provided a new way for the rapid discrimination of the water inrush source. WEN Tingxin et al. [9] established a mine water inrush source identification model based on KPCA-PSO-RBF-SVM by applying Kernel Principal Component Analysis (KPCA) to extract features and optimizing the kernel parameters and penalty factor of Support Vector Machines (SVM) by combining the PSO and Radial Basis Function (RBF), which provides a new way of identifying the water inrush source. LI Lin [10] proposed a Random Forest (RF) mine water inrush source identification method based on feature importance, which is quickly and accurately able to identify water inrush source even in the absence of water source data. JU Qiding et al. [11] established a water inrush source identification model combining PCA and Bayesian Discriminant (Bayes), which improved the safety of Pan’er coal mine and provided a theoretical reference for mine water prevention and control work in similar mines. SONG Libing et al. [12] established a mine water inrush source identification model based on coupled analysis–outlier test–regression filling method–Bayes with Baode mine as an example, and compared the model identification results with that of the PCA-Bayes model, which provided theoretical support for the identification of water inrush sources in Baode coal mine. BI Y S et al. [13] constructed a Fisher’s discriminant analysis model for mine water inrush sources based on fuzzy cluster analysis and factor analysis, tested the accuracy of the model through re-substitution and cross-validation, and compared it with the traditional Fisher’s discriminant analysis model, which showed a higher discriminant accuracy. The above studies have obtained abundant achievements in mine water inrush source identification, but there are still some defects. For example, the BPNN needs to trained several times because of slow convergence speed; ELM overcomes the shortcomings of the BPNN, but there are problems such as random initialization of input weights and overfitting, which need to further optimized; the SVM is only suitable for dealing with small-sample data and susceptible to parameters, and it is more sensitive to noise and Outliers [14]; RF needs to be improved in solving the classification accuracy of unbalanced datasets with long computational runtime and high computational resource consumption; Bsyes is susceptible to samples, resulting in the classification ambiguity of the structure; Fisher discriminant analysis is too dependent on structure and relevance, and greatly influenced by the data. In summary, the relevant research methods need to be further improved.

In view of this, mathematical modeling methods are combined with machine learning methods and artificial intelligence technology to propose a mine water inrush source discrimination model based on KPCA-ISSA-KELM with Zhaogezhuang mine as an example. The correlation and nonlinearity between hydrochemical data of different aquifers increases the difficulty of identifying inrush water sources. Meanwhile, the discrimination efficiency of algorithms is greatly affected by parameter selection. Therefore, the model explores the types of inrush water sources from the perspective of groundwater chemical composition. Combing qualitative and quantitative factors, nonlinear features are analyzed and extracted through KPCA to eliminate the influence of redundant information. In this way, comprehensive impact of water source characteristic indicators on inrush water sources can be better considered. Kernel Extreme Learning Machines (KELM) can predict and classify nonlinear and high-dimensional water samples according to existing water sample information. Therefore, combining with kernel function, ELM can get better generalization ability and stability. Due to the randomness of the kernel parameters and regularization coefficients in KELM, an improved Sparrow Search Algorithm (ISSA) is adopted to optimize KELM and improve its classification accuracy. Finally, the KPCA-ISSA-KELM model is compared with KPCA-SSA-KELM model, KPCA-KELM model, ISSA-KELM model, SSA-KELM model and KELM model to test the discrimination accuracy of the KPCA-ISSA-KELM model. The model is further applied to a coal mine in Shanxi to verify the stability and practicability with a view to providing theoretical references for the water prevention and control of similar mines.

## 2 Theoretical foundation and discriminant model construction

### 2.1 Kernel Principal Component Analysis (KPCA)

KPCA is a nonlinear data processing method based on high-dimensional feature space, which successfully realizes the dimension reduction of linear indivisible datasets by mapping the data in the original space to this space and performing data processing by PCA. Compared with PCA, KPCA is able to capture more sample information and better preserve the local structural information of the data, thus providing a more accurate feature representation. Its specific implementation steps are as follows [15]:

1. KPCA maps the raw water chemistry characterization data to the high-dimensional space *φ*, forming new data *φ*(*e*_*i*_) = [*φ*(*e*_1_), *φ*(*e*_2_),…, *φ*(*e*_*n*_)], *i* = 1,2,…,*n*. Assuming that the samples in the high-dimensional space have shown a trend of centralization, the covariance matrix is as follows:
$$S=\frac{1}{n}\sum_{i=1}^{n}\varphi (e_{i})\varphi {\left(e_{j}\right)}^{T}=\frac{1}{n}\varphi \varphi^{T}$$ (1)
2. By introducing the kernel function K* = *φ*^T^*φ*, the data in *S* is solved by principal component analysis:
$$K^{*}\zeta =\lambda \zeta$$ (2)
  Where: *λ* is the eigenvalue; *ζ* is the eigenvector.
3. The cumulative contribution rate is set as 85%, descend order and take the first *m* eigenvalues *λ*_*j*_(*j* = 1,2,…,*m*) with their corresponding eigenvectors *ζ*_*j*_(*j* = 1,2,…,*m*):
$$\frac{\sum_{j=1}^{m}\lambda_{j}}{\sum_{i=1}^{m}\lambda_{i}}\geq 85\%$$ (3)
4. The nonlinear samples *H* from the dimension reduction mapping are counted when the cumulative contribution rate meets the set requirements:
$$H={\left[\sum_{i=1}^{n}\zeta_{i}\varphi (e_{i})\right]}^{T}=\zeta^{T}{\left[\varphi (e_{1},e),\cdots ,\varphi (e_{i},e)\right]}^{T}$$ (4)

### 2.2 Kernel extreme learning machine

KELM is an improved algorithm by combining ELM and kernel function proposed by HUANG et al. [16] in 2012. ELM is a new fast learning method based on feed-forward neural network, with less parameter adjustment, that is, it only need to set the number of nodes in the hidden layer and randomly generate the input weights and bias of the hidden layer without iteration, which can obtain the best solution. And KELM not only retains the advantages of ELM such as fast training speed and strong nonlinear fitting ability, but also effectively improves the robustness, stability, and generalization of ELM, especially when dealing with large-scale datasets, it shows better performance [23]. Kernel functions usually have a certain regularization effect, thus limiting the complexity of the model, which is crucial for avoiding overfitting. The traditional ELM calculation process is as follows [17]:

η(x)=h(x)β (5)

$$W=h(x)={\left[\begin{array}{ccc} G(\omega_{1}x_{1}+b_{1}) & \cdots & G(\omega_{k}x_{1}+b_{k})\\ \vdots & \cdots & \vdots \\ G(\omega_{1}x_{N}+b_{1}) & \cdots & G(\omega_{k}x_{N}+b_{k}) \end{array}\right]}_{N\times k}$$ (6)

$$\beta^{*}=W^{+}X$$ (7)

$$W^{+}=W^{T}{\left(WW^{T}\right)}^{-1}$$ (8)

Where: *N* is the number of samples; *ω* is the weight between the input layer and the hidden layer; *b* is the bias of the hidden layer; *W* is the hidden layer matrix of the neural network; *W*^+^ is the generalized inverse matrix of *W*; *β* is the output weight between the hidden layer and the output layer; *G*() is the activation function; *k* is the number of neurons in the hidden layer, and *X* is the vector of the predicted target values.

In the KELM model, the RBF kernel function is used as the kernel function in order to realize the conversion from stochastic mapping to stable kernel mapping. The parameter *I*/*C* is added to the unit diagonal array (*WW*^T^) to prevent characteristic root from being 0. Its specific expression is as follows [18]:

$$\eta (x)=h(x)W^{T}{\left(\frac{I}{C}+WW^{T}\right)}^{-1}X=[\begin{array}{c} g(x,x_{1})\\ \vdots \\ g(x,x_{N}) \end{array}]{\left(\frac{I}{C}+WW^{T}\right)}^{-1}X$$ (9)

$$g(x_{i},x_{j})=\exp(-\gamma ||x_{i}-x_{j}|{|}^{2}),\;\gamma >0$$ (10)

Where: *I* is the N-order unit array; *C* is the regularization factor; *g*(*x*_*i*_,*x*_*j*_) is the kernel function; and *γ* is the kernel parameter coefficient.

### 2.3 Improved sparrow search algorithm

#### 2.3.1 Sparrow search algorithm

Sparrow Search Algorithm (SSA) is an intelligent optimization algorithm based on social features proposed by XUE Jiankai in 2020 [19, 20]. Compared with traditional intelligent optimization algorithms, SSA is characterized with simple in structure, easy to realize, fewer control parameters and better convergence. Sparrow species in SSA are divided into three types: discoverers, joiners and scouts [21].

In the process of predation, discoverers generally undertake the important task of finding food and providing information about the predation area for the population with high fitness values, whose position is updated as follows:

$$X_{i,j}^{t+1}=\{\begin{array}{l} X_{i,j}^{t}\cdot \exp(\frac{-i}{\alpha \cdot iter_{\max}}),\;R_{2}<ST\\ X_{i,j}^{t}+Q\cdot L,\;R_{2}\geq ST \end{array}$$ (11)

Where: *Xt i*,*j* denotes the position of the *ith* sparrow in the *jth* (*j*∈[1,d]) dimension in the *tth* iteration; *iter*_max_ is the maximum number of iterations; *Q* denotes a set of random numbers conforming to a standard normal distribution; *R*_2_ (*R*_2_∈[0,1]) and *ST* (*ST*∈[0.5,1]) denote the alarm value and the safety threshold; *L* stands for the 1×*d* matrix, where each element of the matrix are all 1.

The joiners are usually watching the finders and feeding around them to get better food. The joiners’ location updates are described as follows:

$$X_{i,j}^{t+1}=\{\begin{array}{l} Q\cdot \exp(\frac{X_{worst}^{t}-X_{i,j}^{t}}{i^{2}}),\;i>\frac{n}{2}\\ X_{P}^{t+1}+|X_{i,j}^{t}-X_{P}^{t+1}|\cdot A^{+}\cdot L,\;i\leq \frac{n}{2} \end{array}$$ (12)

Where: *X*_worst_ represents the location of the individual with the worst fitness value; *X*_p_ represents the location of the individual with the optimal fitness value; and *A* represents a 1×*d* matrix with each element of the matrix randomly assigned a value of 1 or -1 and A^+^ = A^T^(AA^T^)^-1^.

The primary task of scouts is to scout for early warning. Scouts provide early warning and engage in anti-predator behavior when sparrow populations are threatened by predators. Their position is updated as follows:

$$X_{i,j}^{t+1}=\{\begin{array}{l} X_{bset}^{t}+\beta \cdot |X_{i,j}^{t}-X_{best}^{t}|,\;f_{i}>f_{g}\\ X_{i,j}^{t}+K\cdot (\frac{\left|X_{i,j}^{t}-X_{worst}^{t}\right|}{(f_{i}-f_{w})+\varepsilon }),\;f_{i}=f_{g} \end{array}$$ (13)

Where: *X*_best_ represents the global optimal position; *K* (*K*∈[–1,1]) is a random number; *β* is a set of random numbers obeying a normal distribution with mean 0 and variance 1; *f*_i_ is the current fitness value of the individual sparrow; *f*_g_ and *f*_w_ denote the current fitness values of the global best and worst individuals, respectively; and *ε* is set to a non-zero value.

#### 2.3.2 Algorithm improvement

As a common problem in machine learning models, the overfitting will occur because of complex model, limiting train data and noise interference. Therefore, SSA is used to optimize the hyperparameters of KELM. However, since the individual sparrows in the SSA achieve convergence by direct jumps to the neighborhood of the current optimal solution, which leads to low convergence accuracy, it is easy to fall into the problems of local optimum and slowing of convergence at the later stage of iteration, and ultimately it is difficult to achieve the global optimal solution. Therefore, three strategies are used to improve the SSA to enhance its global search capability and stability, simultaneously avoiding overfitting in KELM.

1. Use Sine chaotic mapping strategy to initialize the population. The intelligent optimization algorithm uses random generation in the initialization process with poor ergodicity, resulting in the reduction of the quality of the initial solution [22]. The fitness function value of the random number generated by the chaotic mapping is obviously improved and the distribution is more uniform, which makes the subsequent search range wider and helps to improve the accuracy and stability of the algorithm to have better global search capabilities. Sine map is a typical representative of chaotic map with simple form and easy to implement, its specific formula is as follows [23]:
$$x_{k+1}=\frac{1}{4}\sin(\pi x_{k}),\;a\in (0,4]$$ (14)
  Where: *x*_*k*_ is the number of chaos for the *kth* iteration.
2. Introduce dynamic adaptive weights. In order to avoid the algorithm falling into the dilemma of local optimal solution, the population can adjust adaptively during the search process. The global optimal solution of the previous generation is introduced when updating the formula of the discoverer’s position, thus effectively improving the global search ability of the algorithm and avoiding the occurrence of overfitting. In addition, the dynamic weight coefficient ω is introduced into the position update mode of the discoverer by drawing on the idea of inertial weight, so that it can better carry out global exploration [24]. The specific formula is as follows:
$$\omega =\frac{e^{2(1-t/iter_{\max})}-e^{-2(1-t/iter_{\max})}}{e^{2(1-t/iter_{\max})}+e^{2(1-t/iter_{\max})}}$$ (15)
$$X_{i,j}^{t+1}=\{\begin{array}{l} (X_{i,j}^{t}+\omega (f_{j,g}^{t}-X_{i,j}^{t}))\cdot rand,\;R_{2}<ST\\ X_{i,j}^{t}+Q,\;R_{2}\geq ST \end{array}$$ (16)
  Where: $f_{j,g}^{t}$ is the global optimal solution for the *jth* dimension in the previous generation.
3. Integrate Cauchy variation with the reverse learning strategy. In order to enable sparrow individuals to better seek excellence, the reverse learning strategy is integrated into SSA, which is mathematically characterized as follows [25]:
$$X_{best}^{'}(t)=ub+r⊕(lb-Xbest(t))$$ (17)
$$X_{i,j}^{t+1}=X_{best}^{'}(t)+b_{1}⊕(X_{best}(t)-X_{best}^{'}(t))$$ (18)
  Where: $X_{best}^{'}(t)$ is the optimal solution reverse solution by the *tth* iteration; *ub* and *lb* are the upper and lower boundaries; *r* is a random matrix subject to the (0,1) standard uniform distribution; *b*_1_ is the information exchange control parameter, and its formula is the following:
$$b_{1}={\left(iter_{\max}-\frac{t}{iter_{\max}}\right)}^{t}$$ (19)
  In order to further improve the optimization performance of the algorithm, a dynamic selection strategy is adopted. The real-time update of the target position is realized with a certain probability by alternately using the reverse learning strategy and the Cauchy variation perturbation strategy [26]. As for the strategy to update the target position, it is determined by the selection probability *P*_s_, which is calculated as follows [27]:
$$P_{s}=-\exp{\left(1-\frac{t}{iter_{\max}}\right)}^{20}+\theta$$ (20)
  Where: *θ* is the adjustment parameter and its value can be taken as 0.05.

#### 2.3.3 Algorithm verification

**Fig 1:**
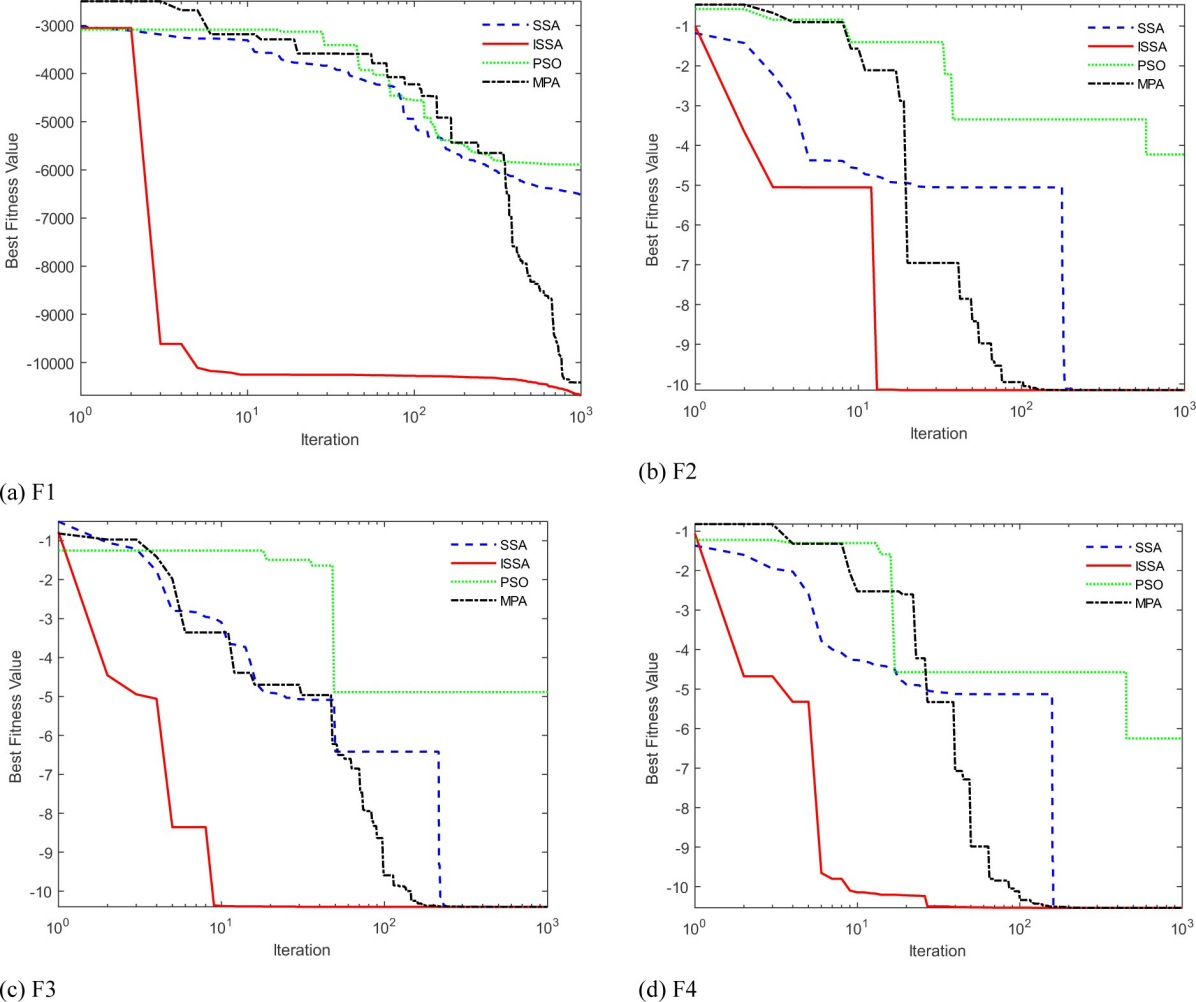
Fitness value iteration curve.

In order to verify the optimization performance of ISSA, four different test functions are used for iterative tests in Matlab2021a environment. ISSA is compared with SSA, PSO algorithm and Marine Predator Algorithm (MPA). The population number and maximum iteration number of each algorithm are uniformly set to 30 and 1000. The proportion of discoverers, participants and scouts in ISSA and SSA is consistent. The optimal fitness value iteration curves of SSA, ISSA, PSO and MPA to test functions F1, F2, F3 and F4 are shown in Fig 1.

According to Fig 1, ISSA has better convergence performance and convergence speed, and most of the ISSA curve is below SSA, PSO and MPA curves, indicating that the solution of the former not only is more accurate than that of SSA, PSO and MPA, but also has better global optimization ability.

### 2.4 KPCA-ISSA-KELM model construction

**Fig 2:**
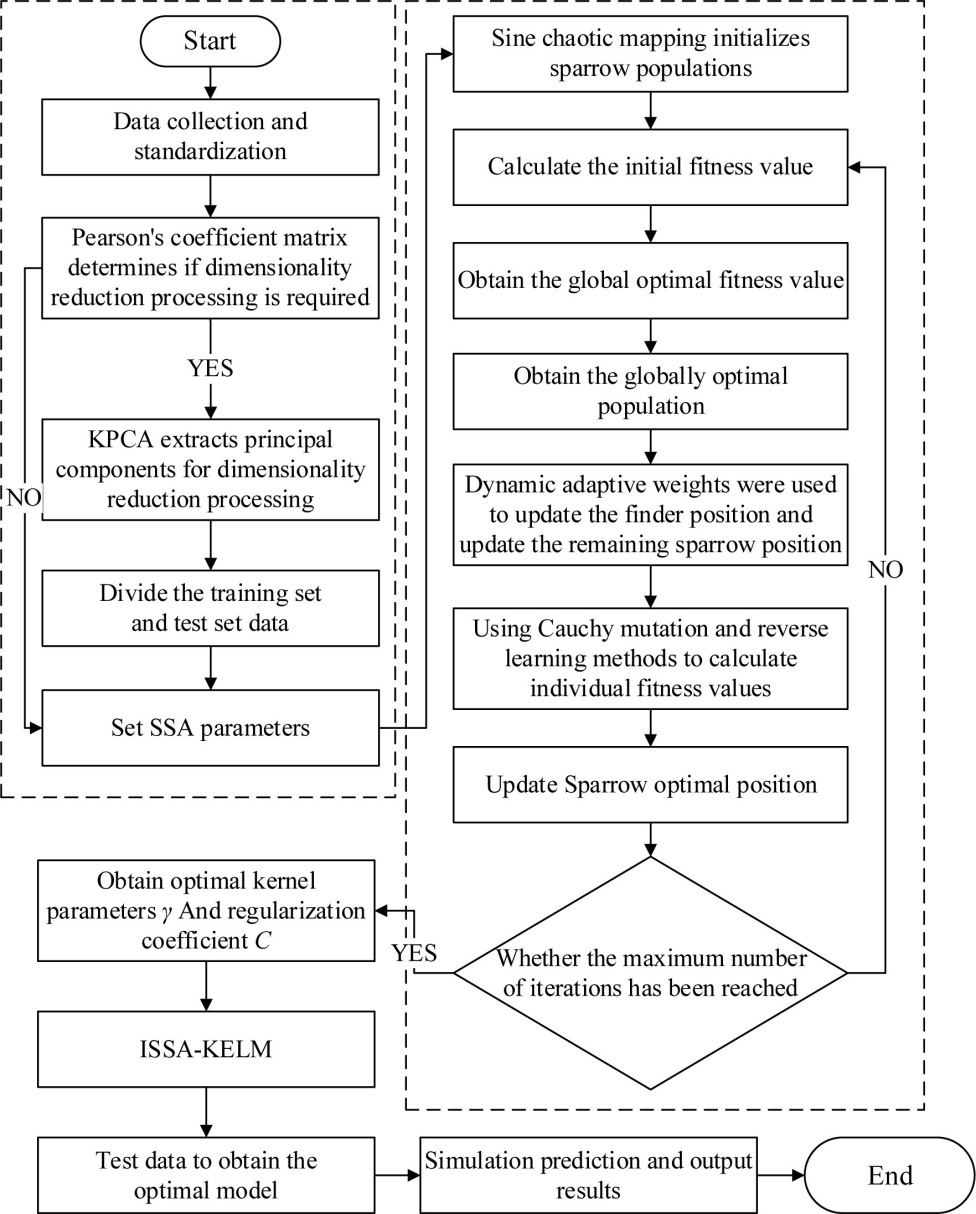
KPCA-ISSA-KELM model establishment process.

In order to reduce the influence of the redundancy of information between indicators on the model discrimination accuracy, KPCA is used to reduce the dimension of the collected data. For the problem that it is not easy to determine the nuclear parameter γ and the regularization coefficient *C* in the KELM, a smaller kernel width or gamma value can increase the generalization ability of the model, thereby further reducing the risk of overfitting. ISSA is used to optimize its hyperparameters. Therefore, a mine water inrush source discrimination model is constructed based on KPCA-ISSA-KELM. The construction process is shown in Fig 2. The specific construction steps are as follows:

1. Standardize the collected data, use Pearson coefficient matrix to determine whether KPCA reduces the dimensionality of the original data, and divide the training and testing sets.
2. Set the relevant parameters of SSA, while use Sine chaos mapping for population initialization.
3. Obtain the initial fitness value, get the global optimal fitness value to find the global optimal population.
4. Introduce dynamic adaptive weights to update the position of the sparrow discoverer, while update the remaining sparrow positions.
5. Calculate the individual fitness values by the Cauchy variation and reverse learning strategies and obtain the optimal parameters for the current position update the position of the sparrow.
6. Determine whether the termination conditions are met. If not, repeat steps (3) to (5). If satisfied, output the optimal kernel parameters *γ* and regularization coefficient *C* to construct ISSA-KELM model for simulation prediction.

## 3 Verification and analysis of water source discrimination model

### 3.1 General situation of mine and selection of discriminant index

**Fig 3:**
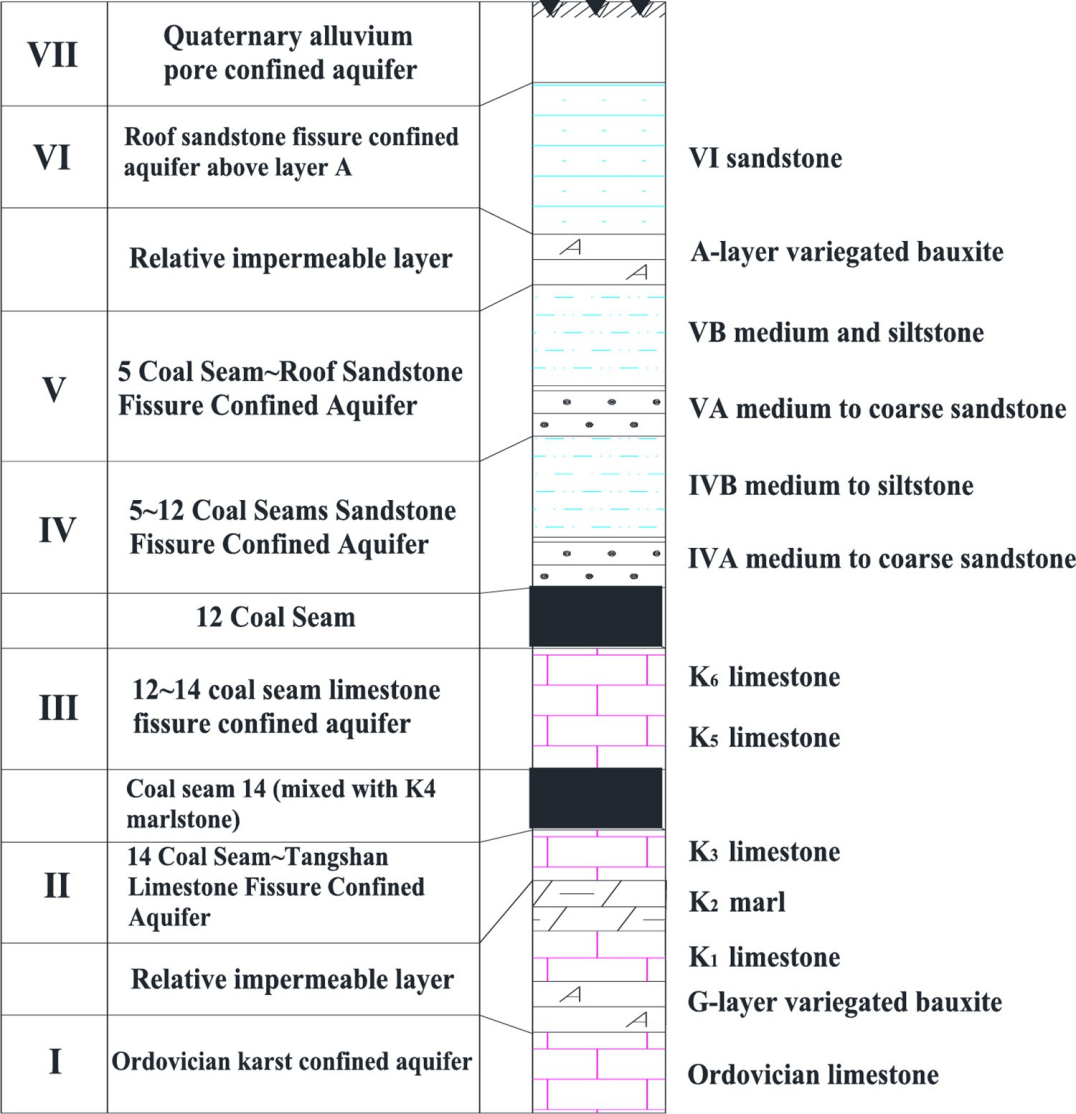
Vertical diagram of aquifer.

Zhaogezhuang Coal Mine in Tangshan Kailuan Mining District, located in the eastern part of the north wing of the syncline of Kaiping Coalfield, is mainly mining lignite and anthracite. The geological structure is dominated by the thrust tectonics, which is one of the mines with the most complicated geological conditions in Kailuan Mining District. The aquifers from bottom to top are as follows: 14 coal seam ~ Tangshan limestone sandstone fissure confined aquifer and Ordovician karst confined aquifer, 12 ~ 14 coal seam limestone fissure confined aquifer, 5 ~ 12 coal seam sandstone fissure confined aquifer, 5 coal seam ~ roof sandstone fissure confined aquifer, roof sandstone fissure confined aquifer above the A layer, and Quaternary alluvial pore confined aquifer. The distribution of specific aquifers is shown in Fig 3.

Four kinds of aquifer water samples with a total of 74 groups were selected from the references [28, 29], namely, the Goaf water, the Austrian gray water, the sandstone fracture water of the 13 coal seam, and the sandstone fracture water of the 12 coal seam. Groups 1–50 are training samples and groups 51–74 are test samples. Referring to the research results of the references [1, 30], six water chemistry characteristic indexes, Na^+^(X1)、Ca^2+^(X2)、Mg^2+^(X3)、Cl^-^(X4)、SO2- 4(X5) and HCO- 3(X6), were selected as the discriminant indexes of the model. Some of the measured data are shown in Table 1.

**Table 1:** Some measured data of Zhaogezhuang coal mine.

NO.	Concentration of each ionic substance/(mg·L-1)						Type of water sample
	X1	X2	X3	X4	X5	X6	
1	11.05	66.34	22.23	1.5	81.28	17.21	1
2	4.36	54.83	40.66	2.68	66.49	30.83	1
3	10.7	54.64	34.18	11.31	70.14	17.54	1
4	5.86	48.45	45.06	1.79	81.84	16.37	1
⋮	⋮	⋮	⋮	⋮	⋮	⋮	⋮
36	15.11	47.81	36.85	8.4	13.81	77.49	3
37	8.08	60.91	31.01	8.56	15.44	72.94	4
38	8.43	59.97	31.55	8.95	16.16	71.8	4
39	8.16	58.08	33.76	8.87	14.91	72.26	4
⋮	⋮	⋮	⋮	⋮	⋮	⋮	⋮
71	9.6	60.39	30.01	8.65	16.38	71.43	4
72	8.43	58.21	30.85	8.7	16.12	71.22	4
73	9.27	57.23	30.5	8.77	15.92	70.8	4
74	9.59	71.73	23.17	17.99	52.65	244.62	4

Note: Goaf water is recorded as 1, Austrian gray water is recorded as 2, 13 coal seam sandstone fissure water is recorded as 3, and 12 coal seam sandstone fissure water is recorded as 4.

### 3.2 Data correlation and KPCA dimension reduction

**Fig 4:**
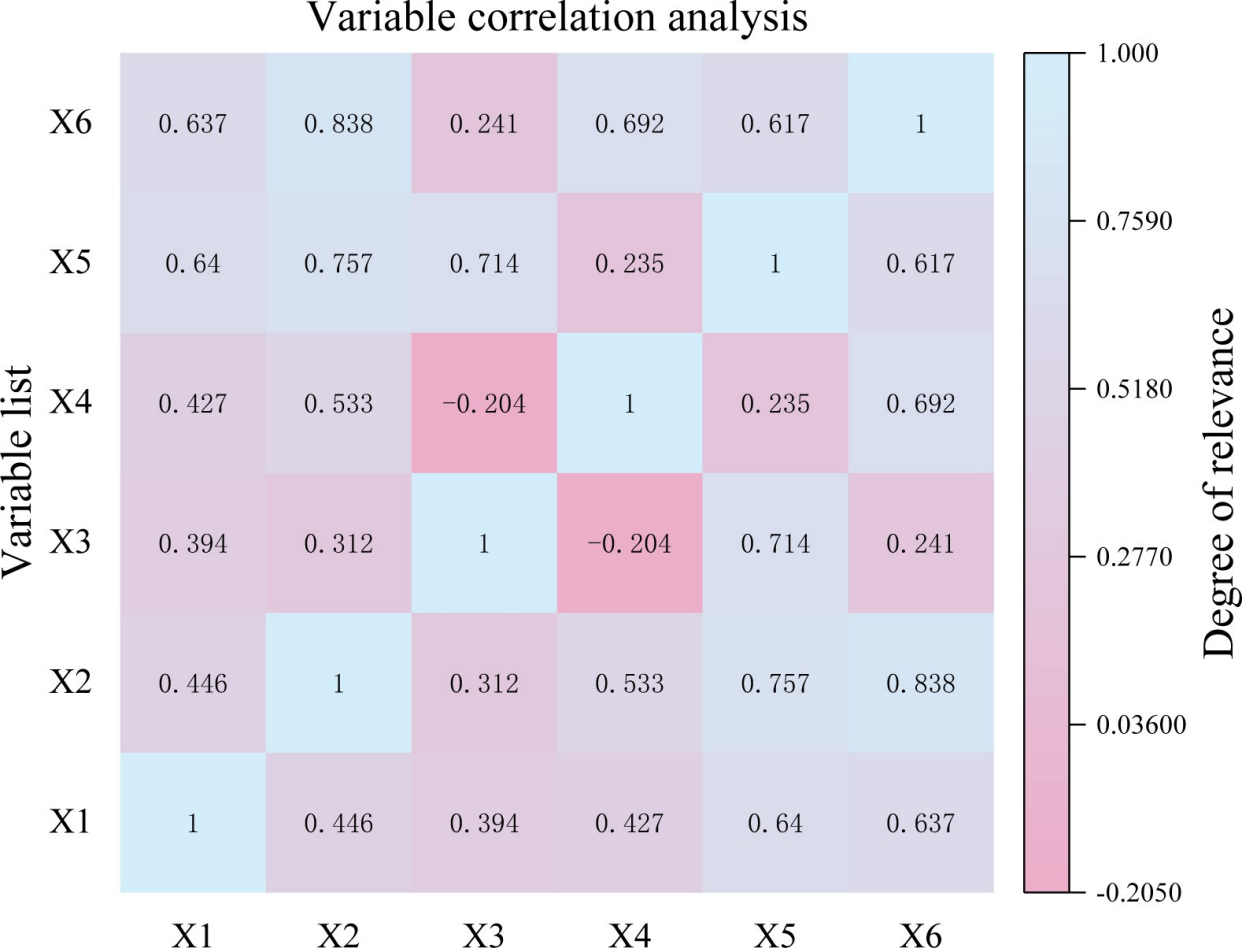
Correlation coefficient matrix of discriminant index.

Due to the large variability of the sample data of mine inrush water sources, it is necessary to standardize the selected data first, and then use SPSS27 software to carry out correlation analysis to derive the Pearson’s correlation coefficient matrix between the indicators, as shown in Fig 4. As can be seen from Fig 4, the correlation coefficients between X2 and X6, X3 and X4, X3 and X6, X3 and X4, and X4 and X5 are 0.838, -0.241, 0.241, and 0.235, respectively. When the correlation coefficient between the two indicators is less than 0.3, it indicates that they may be of little help to information enrichment and need to be deleted. When the correlation coefficient is greater than 0.8, it indicates that there is data redundancy. If it is used directly, it will inevitably affect the discrimination accuracy to some extent. Therefore, it is necessary to reduce the dimension of the discriminant index to improve its accuracy and reliability.

**Fig 5:**
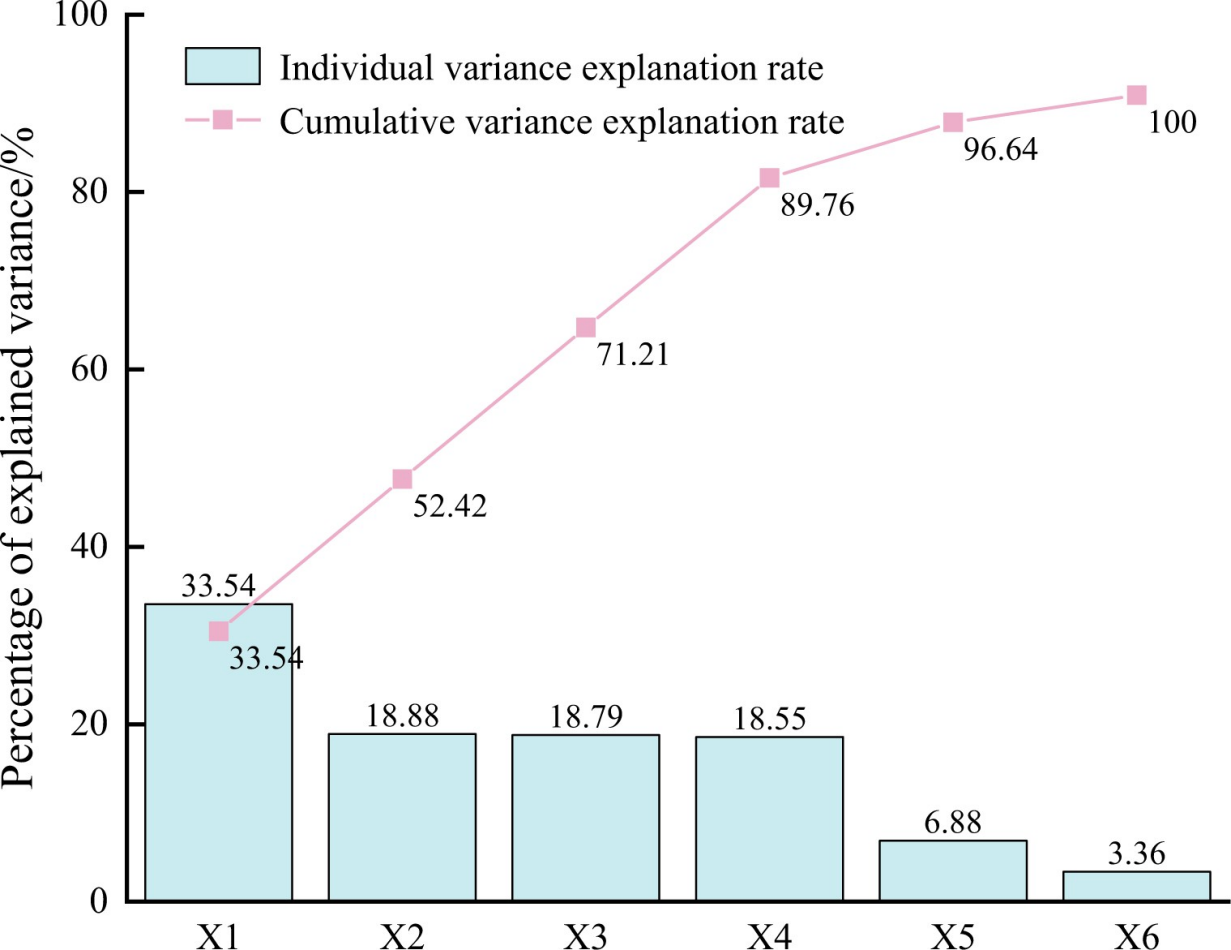
The cumulative proportion of interpretation.

The original data was performed dimension reduction by KPCA with a cumulative variance interpretation exceeding 85% as the selection criterion. Finally, four principal components were extracted, denoted as Y1, Y2, Y3, and Y4, with corresponding variance interpretation rates of 33.54%, 18.88%, 18.79%, and 18.55%, respectively. The cumulative explanatory variance was 89.76%, indicating that the extracted four principal components can satisfactorily reflect the majority of information in the original six hydrochemical indicators. The partial data after dimension reduction is shown in Table 2, and the cumulative proportion of feature interpretation is shown in Fig 5.

**Table 2:** KPCA partial data after dimension reduction.

NO.	Principal component				Type of water sample
	Y1	Y2	Y3	Y4	
1	-0.72555	-0.72314	-1.38025	0.211839	1
2	-0.50761	-0.57996	-0.65552	0.151646	1
3	-0.72479	-0.94948	-0.99556	0.375031	1
4	-0.96187	-1.02202	-0.56187	0.144681	1
⋮	⋮	⋮	⋮	⋮	⋮
36	1.005741	1.362376	0.264915	0.338693	3
37	1.137083	1.175066	-0.23893	0.183529	4
38	1.123813	1.158923	-0.2236	0.197921	4
39	1.139525	1.157773	-0.10703	0.192052	4
⋮	⋮	⋮	⋮	⋮	⋮
71	1.12114	1.176501	-0.27964	0.215371	4
72	1.101673	1.185996	-0.20803	0.197201	4
73	1.100276	1.205051	-0.19801	0.215115	4
74	0.186845	2.032539	0.0543	0.132658	4

### 3.3 Experimental environment and modeling parameters

The computer in the experiment is configured with Win10 64bit operating system, Inter(R) Core(TM) i7-8750H CPU @ 2.20GHz processor, GTX 1060 3GB graphics card, 16GB DDR4 running memory. The test environment is MATLAB 2021a. The required parameters for building the model are shown in Table 3.

**Table 3:** Parameters for model construction.

Parameter name	Specific setting
Sparrow population	30
Maximum iterations	100
Sparrow warning value	0.6
Sparrow finder ratio	0.7
Kernel function	RBF kernel function
Kernel parameter and regularization coefficient	Optimized by ISSA and SSA

### 3.4 Comparison and analysis of test results

**Fig 6:**
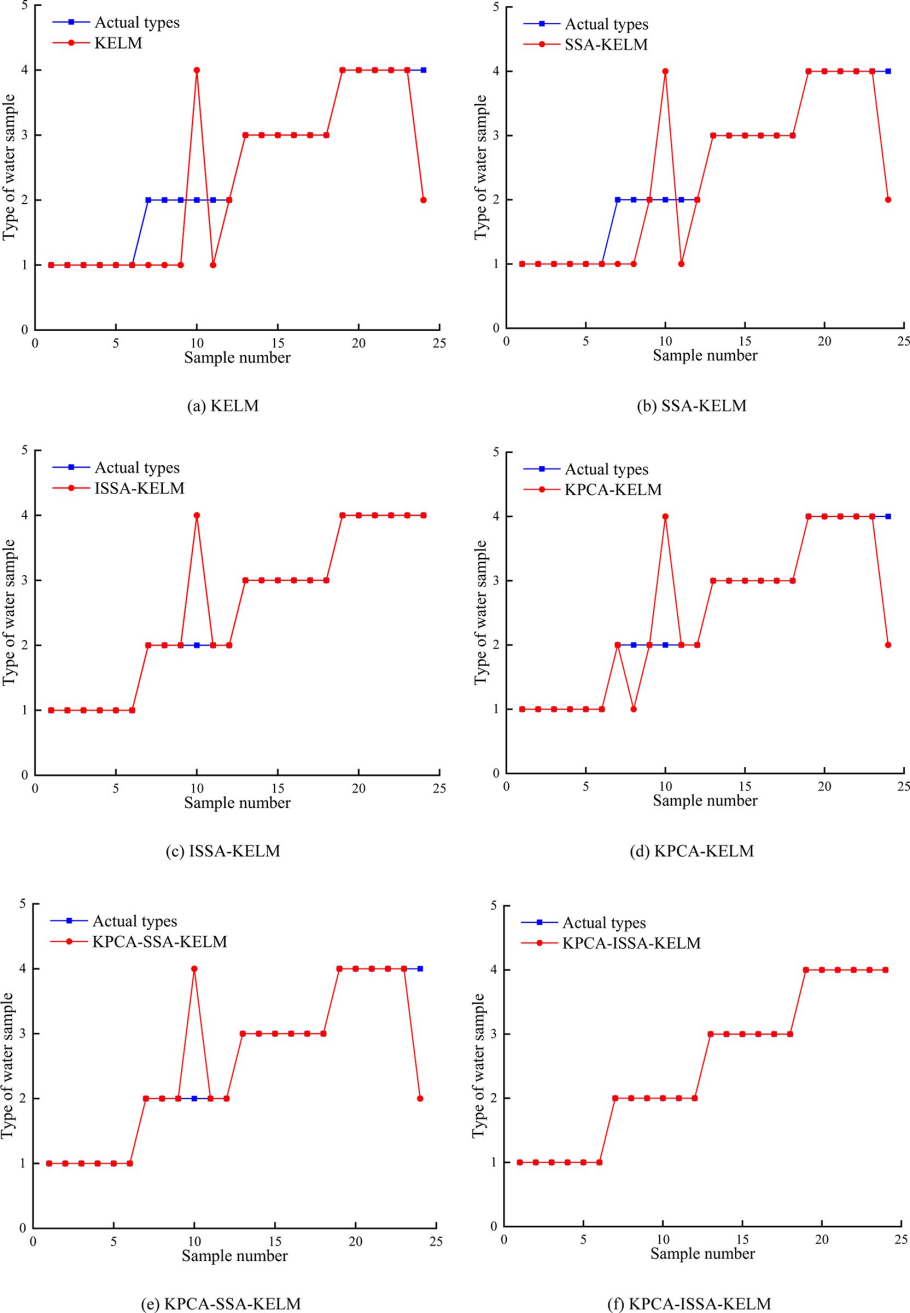
Comparison of the discriminant results of each model.

In order to verify the reliability of the models proposed in the paper, the original data were input into ISSA-KELM, SSA-KELM and KELM models, respectively. The principal component data after dimension reduction were input into KPCA-ISSA-KELM, KPCA-SSA-KELM and KPCA-KELM models for discrimination, respectively. The accuracy of the test samples discrimination results is shown in Table 4. The comparison of the test samples discrimination results of each model is shown in Fig 6.

**Table 4:** The discriminant results of each model test sample.

NO.	Actual types	KELM	SSA-KELM	ISSA-KELM	KPCA-KELM	KPCA-SSA-KELM	KPCA-ISSA-KELM
51	1	1	1	1	1	1	1
52	1	1	1	1	1	1	1
53	1	1	1	1	1	1	1
54	1	1	1	1	1	1	1
55	1	1	1	1	1	1	1
56	1	1	1	1	1	1	1
57	2	1*	1*	2	2	2	2
58	2	1*	1*	2	1*	2	2
59	2	1*	2	2	2	2	2
60	2	4*	4*	4*	4*	4*	2
61	2	1*	1*	2	2	2	2
62	2	2	2	2	2	2	2
63	3	3	3	3	3	3	3
64	3	3	3	3	3	3	3
65	3	3	3	3	3	3	3
66	3	3	3	3	3	3	3
67	3	3	3	3	3	3	3
68	3	3	3	3	3	3	3
69	4	4	4	4	4	4	4
70	4	4	4	4	4	4	4
71	4	4	4	4	4	4	4
72	4	4	4	4	4	4	4
73	4	4	4	4	4	4	4
74	4	2*	2*	4	2*	2*	4
Accuracy rate		75%	79.17%	95.83%	87.50%	91.67%	100%

Note

* indicates that the model predicted water sample type results differed from the actual type.

According to Table 4 and Fig 6, there is highest discrimination accuracy of the KPCA-ISA-KELM model is the highest, reaching 100%, while the discriminant accuracy of KELM, SSA-KELM, ISA-KELM, KPCA-KELM and KPCA-SSA-KELM models are 75%, 79.17%, 95.83%, 87.5% and 91.67%, respectively, indicating that the introduction of Sine chaotic mapping, dynamic adaptive weights, Cauchy variation and reverse learning enhance the optimization ability of SSA, effectively preventing the model from falling into the local optimal solution prematurely, and further improving the generalization ability and prediction accuracy of KELM model.

**Fig 7:**
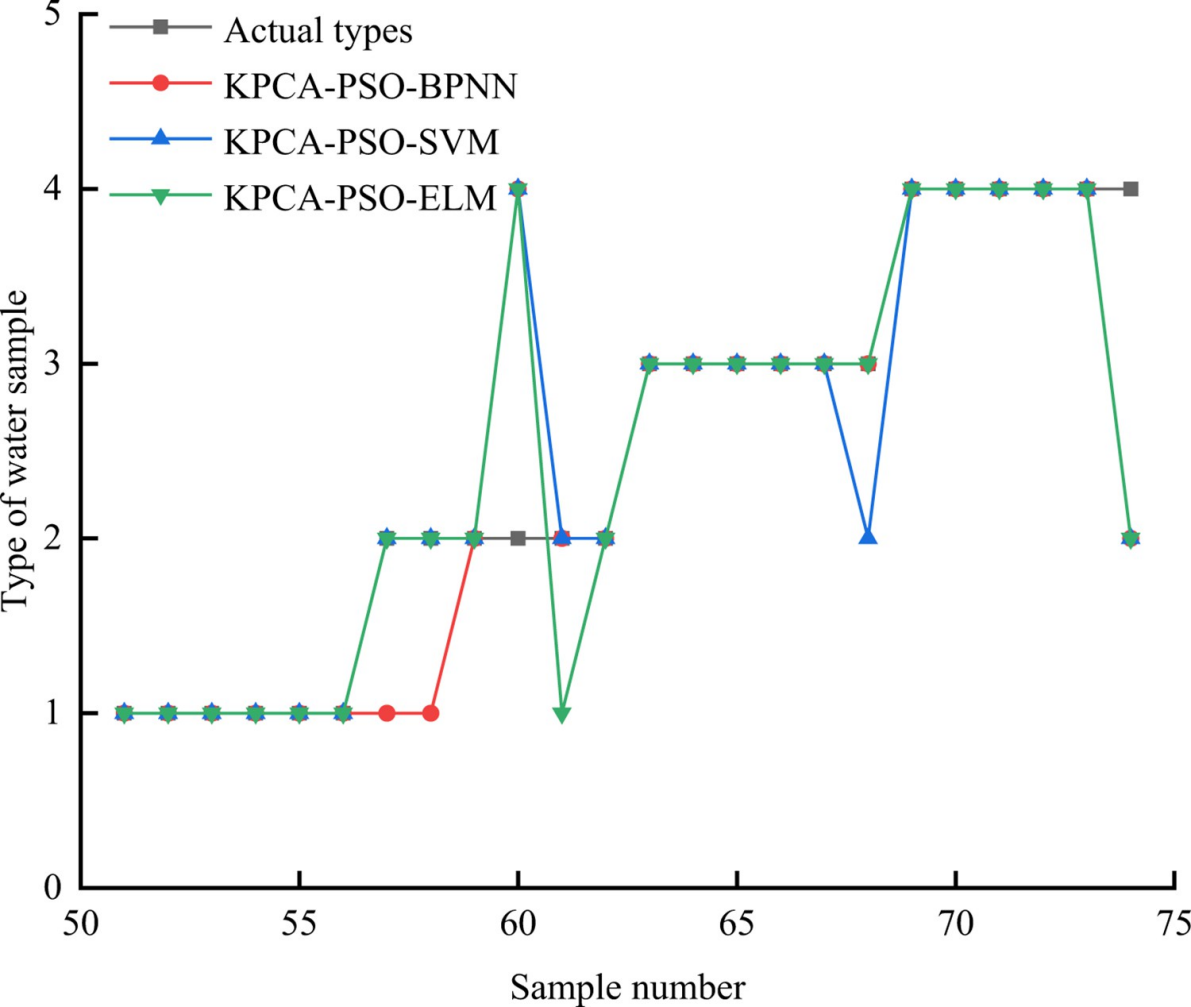
Traditional machine learning models judge results.

Traditional machine learning models BPNN, SVM, and ELM were selected for comparison. On the basis of KPCA dimensionality reduction, the classical intelligent optimization algorithm PSO was used for hyperparameter optimization. The parameter Settings and the discrimination results were shown in Table 5 and Fig 7 respectively. From Fig 7, the discrimination accuracy of the three traditional machine learning models is 83.33%, 87.5%, and 87.5%, respectively, which is significantly lower than the KPCA-ISSA-KELM model constructed by the author, indicating that the KPCA-ISSA-KELM model can more accurately distinguish the source of mine water inrush.

**Table 5:** Traditional machine learning model parameter settings.

Parameter name	Specific setting
Sparrow population	30
Maximum iterations	100
PSO learning factor	1.5
PSO inertia weight	0.8
BPNN training times	1000
BPNN target error	1×10^−6^
BPNN learning rate	0.01

Root mean square error (RMSE), mean absolute ratio error (MAPE), coefficient of determination (*R*^2^), as well as accuracy, recall rate, and F1-score used in classification learning were selected to further objectively compare the discrimination results of various models as evaluation indicators. The discrimination error of each model is shown in Table 6. According to Table 6, the RMSE and MAPE of the KPCA ISSA-KELM model drops significantly compared to other discriminant models, indicating that the model has higher discriminant accuracy and better robustness. Moreover, after using KPCA to reduce the dimensionality of the original data, RMSE and MAPE of the model outperforms data with no dismensionality reduction, indicating that the interference caused by information redundancy can be effectively eliminated by reducing the dimensionality of the data and the classification accuracy of the model is improved. Compared with other models, *R*^2^ significantly improves, indicating a low degree of discretization. Among the three evaluation indicators in classification learning, the KPCA-ISSA-KELM model outperforms other models.

**Table 6:** Error comparison of discriminant results.

Models	RMSE	MAPE/%	*R* ^2^	Precision	Recall	F1-score
KELM	0.7071	14.58	0.6	0.73	0.75	0.74
SSA-KELM	0.677	12.5	0.63	0.79	0.79	0.79
ISSA-KELM	0.4082	4.17	0.87	0.96	0.96	0.96
KPCA-KELM	0.6124	8.33	0.7	0.87	0.88	0.87
KPCA-SSA-KELM	0.5773	6.25	0.73	0.92	0.92	0.92
KPCA- ISSA-KELM	0	0	1	1	1	1

## 4 Examples of engineering applications

The KPCA-ISSA-KELM water source discrimination model is constructed based on the water inrush source data of Zhaogezhuang Coal Mine in Kailuan Mining area. A coal mine in Shanxi Province is selected to examine the stability and practicability of KPCA-ISSA-KELM water source discrimination model. The mine is located in the southeast of the Qinshui Coalfield. The coal seams is characterized by storage stable, a gentle dip, large minable thickness, and abundant reserves. There are perennial rivers on the surface of the area and its periphery. A large number of goafs have been formed after years of mining. The water accumulation in the goaf will have an impact on the next coal seam mining. According to the difference of water chemical characteristics in each aquifer of the mine, the water sources of water inrush can be divided into three types: coal system water, Tai limestone water and Ordovician limestone water. According to its actual situation, the ionic concentration and the total hardness of Na^+^+k^+^, Ca^2+^, Mg^2+^, Cl^-^, SO2- 4 and HCO- 3 are selected as the discriminating indexes of the water sources of water inrush discriminating model. At the same time, 104 groups of measured water inrush data of the mine are compiled as research samples: 31 groups of coal measures water (for short 1), 58 groups of Tai limestone water (for short 2) and 15 groups of Ordovician limestone water (for short 3). 1–83 groups are training samples and 84–104 groups are test samples.

**Fig 8:**
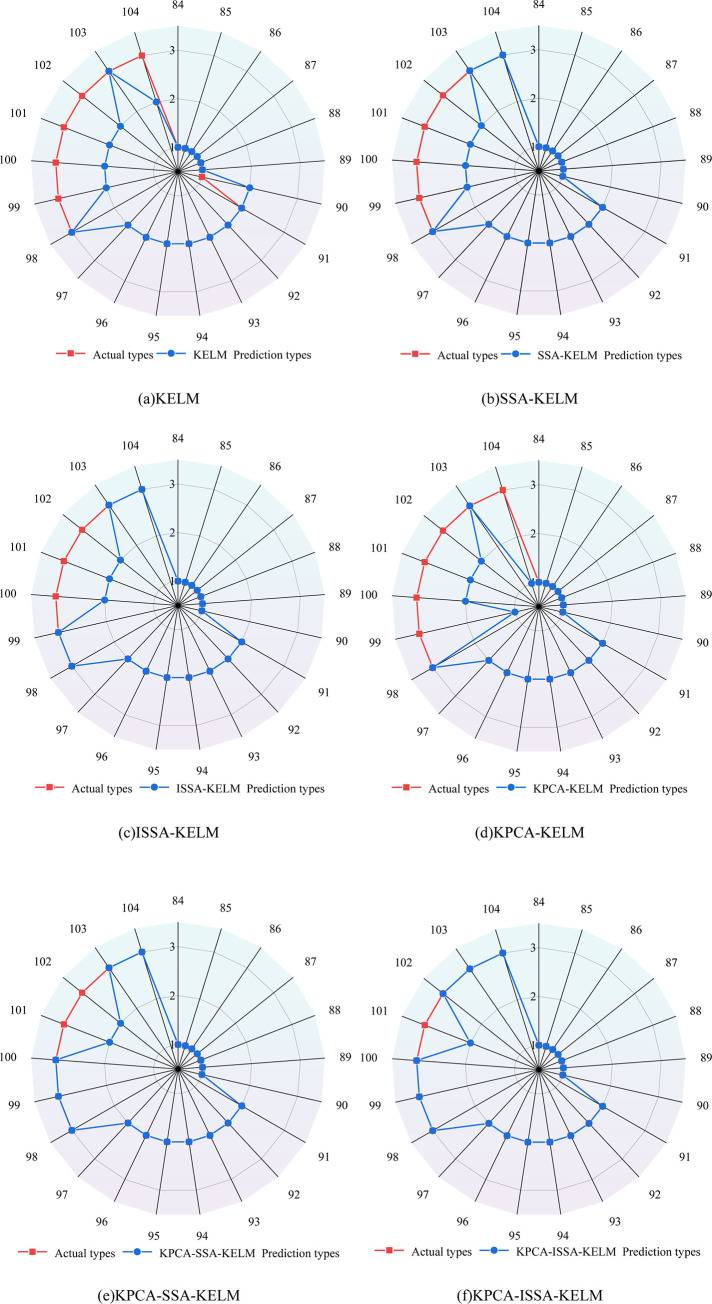
Each model discriminates the result.

**Fig 9:**
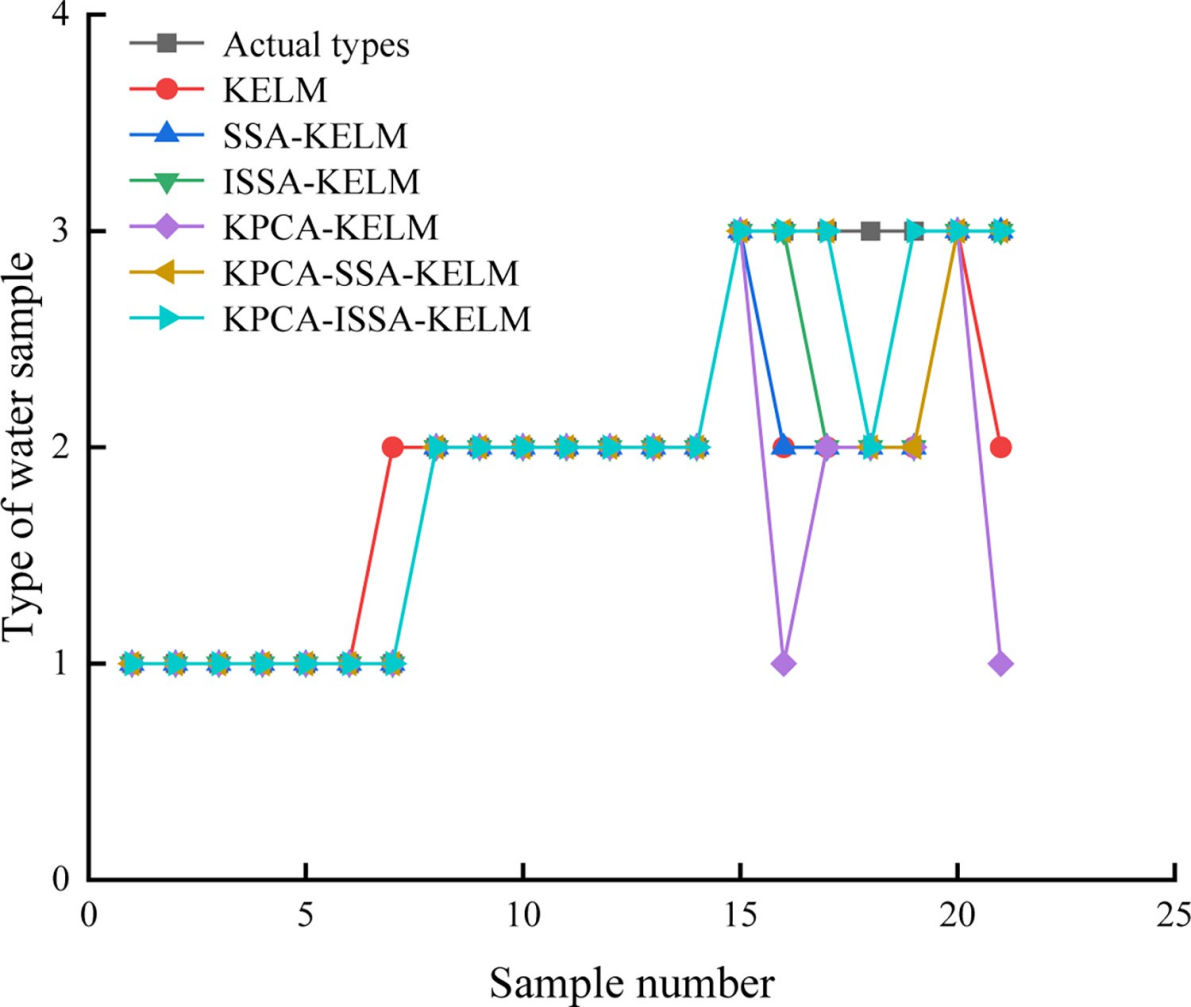
Comparison of discrimination results for water inrush sources.

**Fig 10:**
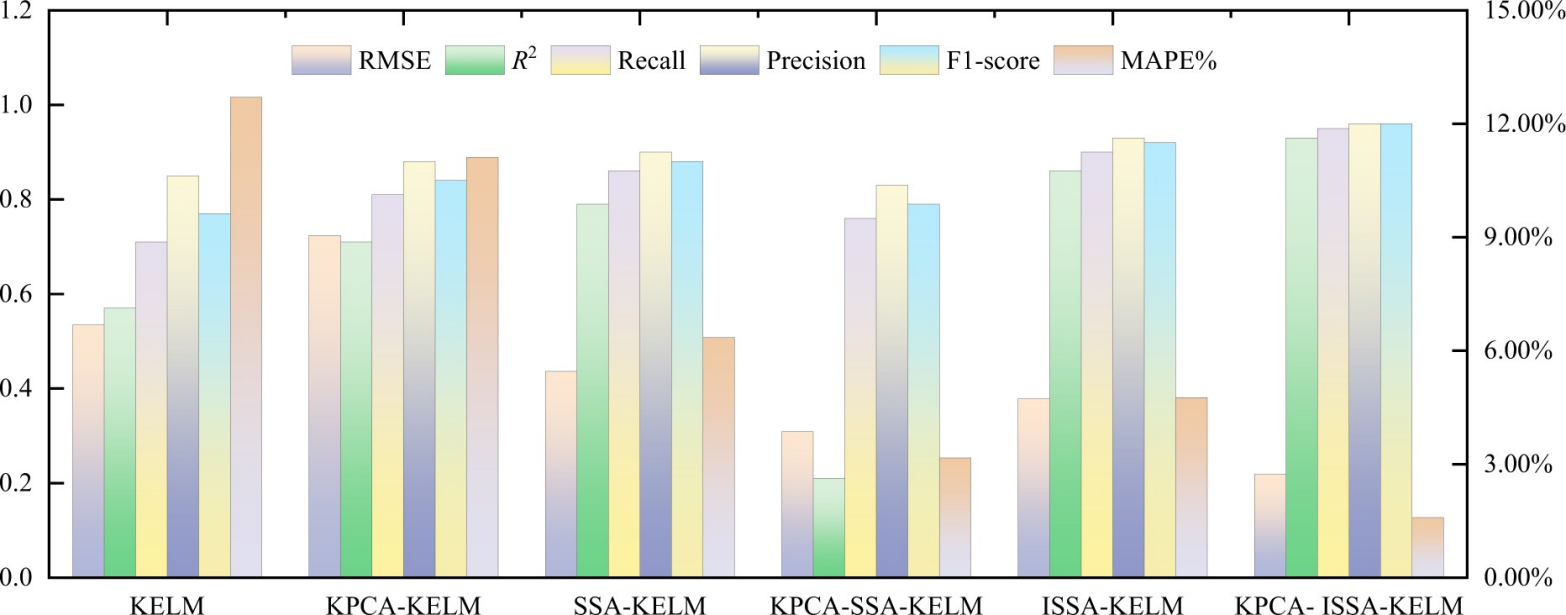
Error comparison of water source discrimination results for water inrush.

The sample data are input into KPCA-ISSA-KELM, KPCA-SSA-KELM, KPCA-KELM, ISSA-KELM, SSA-KELM, and KELM models respectively for discrimination. The discrimination results of each model are shown in Fig 8. The misjudgment rate is shown in Table 7. The comparison of discrimination results is shown in Fig 9. The comparison of discrimination indicators is shown in Fig 10. The parameters of each model are in line with the above.

**Table 7:** Error comparison of discriminant results.

Models	KELM	SSA-KELM	ISSA-KELM	KPCA-KELM	KPCA-SSA-KELM	KPCA-ISSA-KELM
Misjudgment rate	28.57%	19.05%	14.29%	23.81%	9.52%	4.76%

As can be seen from Fig 8 and Table 7, the number of misjudgments in the KELM model is 6, with a misjudgment rate of 28.57%; the number of misjudgments in the SSA-KELM model is 4, with a misjudgment rate of 19.05%; the number of misjudgments in the ISSA-KELM model is 3, with a misjudgment rate of 14.29%; the number of misjudgments in the KPCA-KELM model is 5, with a misjudgment rate of 23.81%; the KPCA- SSA-KELM model has a number of misjudgments of 2 and a misjudgment rate of 9.52%, while the KPCA-ISSA-KELM model has the least number of misjudgments of 1 and its misjudgment rate is only 4.76%. Meanwhile, combining with Table 7, it can be seen that for the first water sample type, only the KELM model made an error in the discrimination, and its discrimination accuracy was 85.71%; in the discrimination of the second water sample type, the accuracy of all the models was 100%; while all the models made an error in the discrimination of the third water sample type, and its discrimination accuracy was 28.57% respectively, 42.86%, 57.14%, 28.57%, 71.43% and 85.71% respectively. It can be seen that the types of errors in the discrimination were mainly concentrated in the third water sample type, and it was judged that it might be caused by the small number of training samples of the third water sample. According to Fig 10, it can be seen that the KPCA-ISSA-KELM model performs optimally in the six indicators of RMSE, MAPE, *R*^2^, Precision, Recall, and F1-score, which indicates that it has the lowest discriminative error and the strongest fitting performance. In summary, the KPCA-ISSA-KLEM model has the advantages of strong generalization ability, good stability, low discretization and high prediction accuracy in the application of sudden water source discrimination.

## 5 Conclusions

1. Correlation analysis and KPCA feature extraction on the hydrochemical indicators of water inrush sources in Zhaogezhuang Coal Mine simplified the discrimination model structure and reduced the impact of information redundancy between ion indicators, thereby improving the training efficiency and discrimination accuracy of the model.
2. After Sine chaotic mapping, dynamic adaptive weights, Cauchy variation and reverse learning are integrated into SSA, the improved SSA have better optimization ability and stability. At the same time, the convergence speed and global search ability of ISSA outweighs that of SSA, PSO and MPA by iterative tests. Then ISSA is used to optimize the kernel parameters and regularization coefficients in KELM, which avoids the shortcomings of random values and improves the prediction accuracy of KELM with strong stability and robustness.
3. Comparing the discrimination results of various models, the discrimination accuracy of KELM, SSA-KELM, ISSA-KELM, KPCA-KELM, KPCA-SSA-KELM, and KPCA-ISSA-KELM models are 75%, 79.17%, 95.83%, 87.5%, 91.67%, and 100%, respectively. At the same time, traditional machine learning models RPNN, SVM, and ELM are used to construct discrimination models, and the classic intelligent optimization algorithm PSO is used to optimize them. The data after KPCA dimensionality reduction is used for discrimination, and the results are significantly lower than the model constructed by the author, verifying the superiority of the model’s performance. Then, six indicators, RMSE, MAPE, *R*^2^, Precision, Recall, and F1-score, are used to objectively compare the discrimination results, verifying from multiple aspects that the KPCA-ISSA-KELM model has higher accuracy and generalization performance compared to other models.
4. A coal mine in Shanxi Province is selected for example application. The results shows that the misjudgment rate of KPCA-ISA-KELM model is the lowest with 4.76%, while that of other models are 28.57%, 19.05%, 14.29%, 23.81% and 9.52%, respectively. The practicability and stability of KPCA-ISA-KELM model are further verified, which can meet the needs of engineering practice and provide a new way for the rapid and accurate identification of water inrush source.
5. Considering the complex hydrogeological conditions of the mining area, the differences in geological conditions of different mining areas, and the influence of human activities, it is necessary to widely collect data from different periods and different mining areas in the future to enhance the university of the model.

## Supporting information

S1 File Zhaogezhuang coal mine in Tangshan Kailuan Mining District data. (DOCX)

S2 File Dimensionality reduction data of Zhaogezhuang coal mine. (DOCX)

S3 File Data of a coal mine in Shanxi. (DOCX)

S4 File Dimension reduction data of a coal mine in Shanxi Province. (DOCX)
