# Supplementary material for: Mine water inrush source discrimination model based on KPCA-ISSA-KELM
Source: PLoS One. 2024 Jun 3;19(6):e0299476. doi: 10.1371/journal.pone.0299476 (PMC11146743; doi:10.1371/journal.pone.0299476)
Supplement: S1 File — (DOCX) [file pone.0299476.s001.docx]

| **Zhaogezhuang Coal Mine in Tangshan Kailuan Mining District data** | | | | | | |
| --- | --- | --- | --- | --- | --- | --- |
| **Na^+^** | **Ca^2+^** | **Mg^2+^** | **CL^-^** | **SO2- 4** | **HCO- 3** | **Type of water sample** |
| 11.05 | 66.34 | 22.23 | 1.5 | 81.28 | 17.21 | 1 |
| 4.36 | 54.83 | 40.66 | 2.68 | 66.49 | 30.83 | 1 |
| 10.7 | 54.64 | 34.18 | 11.31 | 70.14 | 17.54 | 1 |
| 5.86 | 48.45 | 45.06 | 1.79 | 81.84 | 16.37 | 1 |
| 8.91 | 53.43 | 37.46 | 8.45 | 60.47 | 31.08 | 1 |
| 6.05 | 54.13 | 38.71 | 1.81 | 76.57 | 21.62 | 1 |
| 5.69 | 51.7 | 41.83 | 1.65 | 81.41 | 16.85 | 1 |
| 18.02 | 40.9 | 40.82 | 1.75 | 81.83 | 16.25 | 1 |
| 6.95 | 44.44 | 48.09 | 1.44 | 85.55 | 13 | 1 |
| 2.54 | 50.85 | 45.59 | 2.46 | 83.76 | 13.78 | 1 |
| 6.09 | 49.63 | 43.92 | 1.58 | 81.75 | 16.67 | 1 |
| 7.62 | 49.75 | 42.4 | 1.66 | 81.88 | 16.46 | 1 |
| 4.72 | 61.35 | 33.83 | 3.26 | 48.6 | 48.13 | 2 |
| 6.14 | 58.45 | 35.16 | 3.22 | 49.56 | 47.21 | 2 |
| 10.46 | 64.28 | 24.96 | 13.06 | 15.71 | 60.34 | 2 |
| 11.15 | 67.79 | 21.06 | 11.36 | 19.93 | 58.08 | 2 |
| 11.88 | 66.2 | 21.91 | 13.77 | 25.31 | 51.48 | 2 |
| 8.8 | 65.27 | 24.18 | 11.16 | 14.74 | 61.63 | 2 |
| 12.49 | 60.85 | 24.98 | 12.57 | 23.62 | 52.27 | 2 |
| 10.69 | 51.17 | 37.61 | 9.53 | 14.63 | 69.08 | 2 |
| 71.53 | 109.69 | 70.95 | 28.72 | 359.12 | 367.34 | 2 |
| 17.52 | 92.84 | 17.49 | 87.51 | 65.42 | 242.18 | 2 |
| 16.08 | 86.16 | 20.32 | 29.76 | 48.38 | 236.8 | 2 |
| 22.78 | 216.59 | 72.95 | 29.22 | 436.91 | 488.14 | 2 |
| 5.97 | 54.01 | 39.52 | 9.22 | 13.66 | 76.64 | 3 |
| 8.13 | 55.92 | 35.51 | 9.36 | 11.97 | 78.18 | 3 |
| 9.74 | 51.28 | 38.29 | 8.45 | 13.95 | 76.99 | 3 |
| 8.36 | 52.14 | 39.12 | 9.41 | 13.57 | 76.96 | 3 |
| 1.54 | 67.1 | 31.1 | 8.76 | 14.58 | 72.14 | 3 |
| 9.85 | 52.61 | 36.57 | 8.44 | 15.71 | 75.23 | 3 |
| 10.31 | 51.09 | 38 | 8.65 | 14.66 | 76.08 | 3 |
| 9.49 | 52.49 | 37.5 | 8.77 | 12.88 | 77.79 | 3 |
| 7.71 | 55.31 | 36.37 | 8.67 | 13.17 | 77.79 | 3 |
| 9.41 | 50.43 | 38.87 | 8.42 | 14.59 | 76.6 | 3 |
| 12.9 | 50.31 | 36.22 | 9.04 | 12.34 | 78.23 | 3 |
| 15.11 | 47.81 | 36.85 | 8.4 | 13.81 | 77.49 | 3 |
| 8.08 | 60.91 | 31.01 | 8.56 | 15.44 | 72.94 | 4 |
| 8.43 | 59.97 | 31.55 | 8.95 | 16.16 | 71.8 | 4 |
| 8.16 | 58.08 | 33.76 | 8.87 | 14.91 | 72.26 | 4 |
| 12.67 | 51.17 | 35.77 | 10.99 | 12.71 | 74.11 | 4 |
| 7.77 | 57.13 | 34.88 | 9.03 | 13.32 | 74.16 | 4 |
| 8.8 | 57.5 | 33.62 | 9.94 | 15.3 | 70.81 | 4 |
| 4.63 | 58.01 | 37.36 | 10.96 | 17.03 | 69.47 | 4 |
| 3.78 | 61.88 | 34.32 | 5.71 | 16.71 | 73.42 | 4 |
| 9.4 | 59.59 | 30.95 | 9.54 | 15.3 | 71.23 | 4 |
| 2.92 | 61.3 | 35.78 | 9.56 | 15.85 | 71.05 | 4 |
| 7.33 | 60.34 | 32.33 | 8.61 | 16.4 | 70.84 | 4 |
| 11.95 | 56.99 | 30.87 | 9.01 | 14.93 | 73.84 | 4 |
| 6.8 | 62.73 | 30.13 | 8.57 | 16.88 | 70.61 | 4 |
| 5.58 | 64.36 | 29.94 | 8.8 | 16.42 | 71.25 | 4 |
| 7.86 | 48.59 | 43.2 | 1.54 | 82.39 | 16.07 | 1 |
| 11.81 | 49.18 | 37.87 | 3.08 | 71.87 | 21.7 | 1 |
| 12.36 | 52.11 | 34.83 | 2.92 | 72.3 | 22.78 | 1 |
| 11.77 | 50.85 | 36.88 | 2.51 | 78.22 | 19.24 | 1 |
| 10.37 | 49.73 | 39.47 | 2.12 | 83.91 | 13.9 | 1 |
| 9.16 | 50.23 | 40.17 | 2.13 | 82.78 | 15.09 | 1 |
| 6.93 | 57.86 | 39.66 | 4.47 | 53.01 | 41.86 | 2 |
| 6.52 | 59.38 | 40.44 | 5.61 | 51.8 | 41.7 | 2 |
| 5.53 | 60.29 | 38.49 | 4.6 | 50.74 | 44.62 | 2 |
| 5.87 | 64.68 | 29.45 | 8.25 | 20.93 | 68.48 | 2 |
| 6.26 | 58.53 | 39.73 | 5.19 | 49.24 | 43.23 | 2 |
| 24.18 | 102.66 | 25.55 | 37.52 | 95.49 | 268.48 | 2 |
| 12.96 | 48.26 | 38.24 | 9.65 | 13.88 | 76.17 | 3 |
| 9.69 | 52.5 | 37.16 | 9.48 | 10.86 | 79.58 | 3 |
| 12.63 | 47.16 | 39.19 | 9.2 | 14.77 | 75.28 | 3 |
| 14.49 | 46.62 | 37.7 | 9.41 | 13.74 | 76.91 | 3 |
| 13.28 | 49.67 | 37.85 | 10.03 | 12.43 | 77.05 | 3 |
| 12.9 | 47.98 | 30.74 | 17.11 | 34.16 | 221.01 | 3 |
| 5.92 | 61.89 | 31.72 | 8.61 | 15.95 | 70.99 | 4 |
| 8.35 | 58.11 | 33.54 | 8.75 | 15.54 | 71.92 | 4 |
| 9.6 | 60.39 | 30.01 | 8.65 | 16.38 | 71.43 | 4 |
| 8.43 | 58.21 | 30.85 | 8.7 | 16.12 | 71.22 | 4 |
| 9.27 | 57.23 | 30.5 | 8.77 | 15.92 | 70.8 | 4 |
| 9.59 | 71.73 | 23.17 | 17.99 | 52.65 | 244.62 | 4 |
