# Supplementary material for: Mine water inrush source discrimination model based on KPCA-ISSA-KELM
Source: PLoS One. 2024 Jun 3;19(6):e0299476. doi: 10.1371/journal.pone.0299476 (PMC11146743; doi:10.1371/journal.pone.0299476)
Supplement: S2 File — (DOCX) [file pone.0299476.s002.docx]

| **Dimensionality reduction data of Zhaogezhuang coal mine** | | | | |
| --- | --- | --- | --- | --- |
| **Y1** | **Y2** | **Y3** | **Y4** | **Type of water sample** |
| -0.72555 | -0.72314 | -1.38025 | 0.211839 | 1 |
| -0.50761 | -0.57996 | -0.65552 | 0.151646 | 1 |
| -0.72479 | -0.94948 | -0.99556 | 0.375031 | 1 |
| -0.96187 | -1.02202 | -0.56187 | 0.144681 | 1 |
| -0.43169 | -0.53384 | -0.73971 | 0.338909 | 1 |
| -0.78878 | -0.82591 | -0.78579 | 0.15623 | 1 |
| -0.91006 | -0.97659 | -0.69603 | 0.141075 | 1 |
| -1.2095 | -0.984 | -0.54149 | 0.349022 | 1 |
| -1.07891 | -1.11582 | -0.44574 | 0.151125 | 1 |
| -0.92821 | -1.07827 | -0.60475 | 0.097753 | 1 |
| -0.94474 | -1.00339 | -0.60984 | 0.146262 | 1 |
| -0.96335 | -0.99557 | -0.65482 | 0.171311 | 1 |
| 0.166045 | 0.170228 | -0.73187 | 0.143142 | 2 |
| 0.081284 | 0.133266 | -0.65869 | 0.173143 | 2 |
| 1.259092 | 0.968026 | -0.62289 | 0.366946 | 2 |
| 1.150086 | 0.894873 | -0.8711 | 0.354113 | 2 |
| 1.025152 | 0.692974 | -1.00208 | 0.435238 | 2 |
| 1.259206 | 0.986776 | -0.62484 | 0.299973 | 2 |
| 1.039678 | 0.758818 | -0.77963 | 0.454236 | 2 |
| 1.101851 | 1.119287 | 0.144473 | 0.323327 | 2 |
| -1.2635 | 1.240359 | -0.27488 | 0.330254 | 2 |
| 0.197772 | 2.026794 | -0.20213 | 0.476849 | 2 |
| 0.323978 | 2.025514 | -0.08499 | 0.2416 | 2 |
| -0.9245 | 1.252686 | -0.51336 | 0.154285 | 2 |
| 1.088738 | 1.174063 | 0.172547 | 0.18148 | 3 |
| 1.135406 | 1.266587 | 0.05623 | 0.215092 | 3 |
| 1.047812 | 1.254606 | 0.208458 | 0.239761 | 3 |
| 1.07241 | 1.225537 | 0.211322 | 0.226119 | 3 |
| 1.19273 | 1.033115 | -0.34843 | 0.100201 | 3 |
| 1.029448 | 1.235119 | 0.107462 | 0.24669 | 3 |
| 1.036704 | 1.249108 | 0.19558 | 0.255668 | 3 |
| 1.082334 | 1.270738 | 0.173617 | 0.235649 | 3 |
| 1.100612 | 1.244277 | 0.077486 | 0.202012 | 3 |
| 1.022715 | 1.243785 | 0.235603 | 0.235539 | 3 |
| 1.068613 | 1.350468 | 0.203035 | 0.299942 | 3 |
| 1.005741 | 1.362376 | 0.264915 | 0.338693 | 3 |
| 1.137083 | 1.175066 | -0.23893 | 0.183529 | 4 |
| 1.123813 | 1.158923 | -0.2236 | 0.197921 | 4 |
| 1.139525 | 1.157773 | -0.10703 | 0.192052 | 4 |
| 1.125999 | 1.295042 | 0.147121 | 0.304251 | 4 |
| 1.155127 | 1.190232 | -0.02628 | 0.183515 | 4 |
| 1.143287 | 1.147747 | -0.1144 | 0.220995 | 4 |
| 1.115026 | 1.014193 | -0.05376 | 0.159928 | 4 |
| 1.09407 | 1.071629 | -0.17806 | 0.077175 | 4 |
| 1.151363 | 1.175241 | -0.22902 | 0.223512 | 4 |
| 1.149215 | 1.021341 | -0.1429 | 0.108378 | 4 |
| 1.127639 | 1.111532 | -0.22127 | 0.175134 | 4 |
| 1.111567 | 1.27748 | -0.14675 | 0.260839 | 4 |
| 1.129232 | 1.105423 | -0.33508 | 0.163533 | 4 |
| 1.1478 | 1.093617 | -0.35979 | 0.142854 | 4 |
| -0.99079 | -1.01136 | -0.61711 | 0.17296 | 1 |
| -0.84305 | -0.83281 | -0.72857 | 0.286844 | 1 |
| -0.80373 | -0.75876 | -0.86687 | 0.292278 | 1 |
| -0.93688 | -0.87683 | -0.80951 | 0.25962 | 1 |
| -1.03234 | -1.02574 | -0.75016 | 0.215616 | 1 |
| -0.99377 | -1.00678 | -0.73219 | 0.199091 | 1 |
| -0.0241 | -0.16091 | -0.59938 | 0.206776 | 2 |
| 0.057023 | -0.18425 | -0.60399 | 0.214044 | 2 |
| 0.102827 | -0.04278 | -0.63644 | 0.178726 | 2 |
| 1.036766 | 1.007612 | -0.44864 | 0.204604 | 2 |
| 0.122461 | -0.09269 | -0.5707 | 0.205403 | 2 |
| 0.004742 | 1.940296 | -0.2171 | 0.295445 | 2 |
| 1.028703 | 1.303993 | 0.282799 | 0.319104 | 3 |
| 1.117806 | 1.314755 | 0.191958 | 0.241678 | 3 |
| 0.995586 | 1.276492 | 0.322911 | 0.311546 | 3 |
| 1.000166 | 1.3549 | 0.315739 | 0.343765 | 3 |
| 1.082036 | 1.314865 | 0.254975 | 0.323676 | 3 |
| 0.231426 | 2.064859 | 0.33245 | 0.166609 | 3 |
| 1.14554 | 1.096698 | -0.26251 | 0.148313 | 4 |
| 1.125571 | 1.153128 | -0.12265 | 0.195072 | 4 |
| 1.12114 | 1.176501 | -0.27964 | 0.215371 | 4 |
| 1.101673 | 1.185996 | -0.20803 | 0.197201 | 4 |
| 1.100276 | 1.205051 | -0.19801 | 0.215115 | 4 |
| 0.186845 | 2.032539 | 0.0543 | 0.132658 | 4 |
