# Supplementary material for: Mine water inrush source discrimination model based on KPCA-ISSA-KELM
Source: PLoS One. 2024 Jun 3;19(6):e0299476. doi: 10.1371/journal.pone.0299476 (PMC11146743; doi:10.1371/journal.pone.0299476)
Supplement: S3 File — (DOCX) [file pone.0299476.s003.docx]

| **Data of a coal mine in Shanxi** | | | | | | | |
| --- | --- | --- | --- | --- | --- | --- | --- |
| **Ca^2+^** | **Mg^2+^** | **Na^+^+K^+^** | **HCO- 3** | **CL^-^** | **SO2- 4** | **Total hardness** | **Type of water sample** |
| 0.92 | 0.81 | 10.45 | 10.28 | 0.57 | 0.63 | 4.85 | 1 |
| 5.55 | 4.16 | 0.88 | 5.52 | 1.31 | 3.76 | 27.23 | 1 |
| 1.51 | 1.58 | 4.56 | 5.55 | 1.09 | 0.08 | 8.68 | 1 |
| 0.45 | 0.52 | 37.94 | 32.56 | 1.38 | 1.02 | 2.72 | 1 |
| 0.39 | 0.32 | 20.98 | 8.9 | 1.68 | 9.8 | 1.99 | 1 |
| 0.92 | 0.94 | 19.73 | 13.88 | 1.15 | 5.32 | 5.22 | 1 |
| 0.22 | 0.19 | 31.38 | 26.81 | 0.71 | 0.05 | 1.15 | 1 |
| 1.01 | 1.03 | 31.38 | 30.02 | 0.94 | 2.47 | 5.75 | 1 |
| 0.13 | 0.1 | 21.88 | 2.42 | 0.46 | 0.06 | 0.65 | 1 |
| 0.16 | 0.04 | 15.88 | 14.07 | 0.53 | 0.08 | 0.56 | 1 |
| 0.06 | 0.11 | 18.14 | 13.31 | 1.44 | 2.32 | 0.48 | 1 |
| 0.56 | 0.34 | 19.58 | 17.89 | 0.96 | 1.63 | 50.16 | 1 |
| 0.12 | 0.18 | 18.69 | 17.29 | 0.42 | 0.06 | 0.84 | 1 |
| 0.14 | 0.17 | 2.19 | 18.9 | 0.29 | 0.01 | 0.87 | 1 |
| 0.12 | 0.2 | 19.24 | 18.04 | 0.28 | 0.11 | 0.9 | 1 |
| 0.37 | 0.14 | 33.66 | 0.26 | 0.68 | 0.12 | 1.26 | 1 |
| 0.21 | 0.26 | 31.69 | 19.83 | 0.57 | 8.26 | 1.32 | 1 |
| 0.14 | 0.05 | 23.76 | 21.11 | 0.56 | 0.05 | 0.53 | 1 |
| 0.24 | 0.18 | 26.51 | 13.4 | 0.47 | 0.33 | 1.18 | 1 |
| 0.14 | 17.03 | 2.19 | 18.9 | 0.29 | 0.01 | 0.87 | 1 |
| 0.1 | 0.19 | 22.72 | 19.26 | 0.46 | 0.1 | 0.81 | 1 |
| 5.11 | 3.72 | 17.76 | 19.43 | 1.59 | 5.57 | 24.79 | 1 |
| 1.2 | 0.72 | 43.83 | 40.59 | 2.2 | 0.98 | 5.38 | 1 |
| 0.49 | 0.31 | 8.33 | 8.32 | 0.48 | 0.33 | 2.24 | 1 |
| 0.32 | 0.35 | 10.22 | 10.27 | 0.43 | 0.18 | 1.88 | 2 |
| 4.76 | 2.19 | 0.63 | 6.02 | 0.63 | 0.54 | 19.47 | 2 |
| 4.2 | 1.94 | 1.96 | 6.67 | 0.76 | 0.68 | 17.24 | 2 |
| 3.9 | 1.35 | 1.14 | 4.18 | 0.86 | 1.31 | 14.69 | 2 |
| 5.09 | 1.76 | 0.61 | 6.48 | 0.63 | 0.35 | 19.21 | 2 |
| 68.01 | 1.43 | 13.39 | 13.31 | 0.48 | 1.34 | 5.92 | 2 |
| 5.49 | 3.43 | 3.54 | 5.47 | 0.99 | 5.07 | 23.55 | 2 |
| 4.73 | 2 | 1.15 | 6.4 | 0.6 | 0.68 | 18.87 | 2 |
| 51.25 | 4.35 | 12.21 | 1.34 | 34.62 | 0.15 | 155.93 | 2 |
| 7.54 | 4 | 1.86 | 7.26 | 1.15 | 4.99 | 32.36 | 2 |
| 5.82 | 1.94 | 0.65 | 6.87 | 0.94 | 1.12 | 21.76 | 2 |
| 4.88 | 2.27 | 0.61 | 6.35 | 0.8 | 0.33 | 19.26 | 2 |
| 4.91 | 2.09 | 0.77 | 6.35 | 0.99 | 0.43 | 19.62 | 2 |
| 4.66 | 1.13 | 1.27 | 6.46 | 0.79 | 0.81 | 19.04 | 2 |
| 4.28 | 2.47 | 0.92 | 0.63 | 0.78 | 0.51 | 18.93 | 2 |
| 4.07 | 4.45 | 2.66 | 8.62 | 1.14 | 1.42 | 24.17 | 2 |
| 6.91 | 3.84 | 10.04 | 3.31 | 9 | 1.23 | 30.14 | 2 |
| 0.57 | 0.66 | 0.87 | 1.1 | 0.56 | 0.44 | 3.45 | 2 |
| 3.78 | 1.6 | 2.22 | 6.61 | 0.5 | 0.49 | 15.09 | 2 |
| 5 | 2.7 | 1.49 | 6.93 | 1.08 | 1.18 | 21.59 | 2 |
| 1.95 | 1.99 | 4.83 | 6.94 | 1.11 | 0.72 | 11.05 | 2 |
| 4.28 | 2.4 | 1.09 | 6.52 | 0.71 | 0.44 | 18.73 | 2 |
| 2.91 | 2.52 | 2.74 | 6.9 | 0.68 | 0.59 | 15.22 | 2 |
| 0.37 | 0.22 | 10.21 | 10.12 | 0.46 | 0.22 | 1.65 | 2 |
| 3.46 | 3.12 | 3.24 | 7.21 | 1.04 | 1.57 | 18.45 | 2 |
| 3.37 | 1.76 | 2.89 | 7.05 | 0.7 | 0.27 | 14.38 | 2 |
| 3.74 | 2.77 | 1.55 | 5.88 | 1.11 | 1.07 | 18.25 | 2 |
| 0.34 | 0.68 | 10.29 | 9.99 | 0.71 | 0.27 | 2.86 | 2 |
| 0.17 | 0.06 | 12.97 | 10.89 | 0.71 | 0.28 | 0.64 | 2 |
| 1.98 | 1.93 | 4.5 | 6.74 | 1.13 | 0.54 | 10.96 | 2 |
| 0.22 | 0.21 | 12.06 | 11.17 | 0.55 | 0.08 | 1.21 | 2 |
| 3.43 | 2.51 | 3.47 | 7.34 | 1.05 | 1.02 | 16.66 | 2 |
| 1.72 | 1.84 | 4.92 | 6.87 | 0.97 | 0.64 | 9.98 | 2 |
| 3.46 | 1.72 | 2.2 | 6.26 | 0.48 | 0.64 | 14.52 | 2 |
| 6.08 | 4.75 | 4.78 | 6.19 | 0.79 | 8.5 | 30.27 | 2 |
| 1.9 | 3.63 | 9.77 | 9.12 | 0.63 | 5.53 | 15.49 | 2 |
| 4.45 | 2.12 | 0.98 | 5.44 | 0.67 | 0.34 | 18.42 | 2 |
| 1.93 | 1.96 | 10.24 | 8.87 | 0.72 | 3.6 | 10.91 | 2 |
| 3.51 | 2.2 | 1.97 | 5.77 | 0.74 | 0.48 | 16.01 | 2 |
| 4.09 | 2.19 | 1.21 | 6.45 | 0.7 | 0.35 | 17.61 | 2 |
| 6.12 | 6.9 | 4.92 | 7.87 | 1.11 | 8.96 | 36.51 | 2 |
| 4.4 | 2.31 | 0.88 | 6.38 | 0.68 | 0.53 | 18.81 | 2 |
| 4.39 | 2.08 | 1.4 | 6.62 | 0.57 | 0.68 | 18.14 | 2 |
| 7.8 | 5 | 3.69 | 5.52 | 0.8 | 10.15 | 35.92 | 2 |
| 5.82 | 1.94 | 1.08 | 6.87 | 0.94 | 1.12 | 21.76 | 2 |
| 5.43 | 1.03 | 2.87 | 6.28 | 0.72 | 0.7 | 18.12 | 2 |
| 4.09 | 2.19 | 1.21 | 6.45 | 0.69 | 0.35 | 17.61 | 2 |
| 4.67 | 2.19 | 0.88 | 6.58 | 0.75 | 0.04 | 19.21 | 2 |
| 3.56 | 2.27 | 0.08 | 6.58 | 0.69 | 0.5 | 16.87 | 2 |
| 2.96 | 2.36 | 2.68 | 6.27 | 0.95 | 0.78 | 14.92 | 2 |
| 0.2 | 0.1 | 14.06 | 12.33 | 0.68 | 0.23 | 0.84 | 2 |
| 0.32 | 0.48 | 2.15 | 0.9 | 1.15 | 0.03 | 2.24 | 3 |
| 0.21 | 1.03 | 1.41 | 0.89 | 0.9 | 0.02 | 3.48 | 3 |
| 1.29 | 0.43 | 1.75 | 1.38 | 1.5 | 0.48 | 4.8 | 3 |
| 0.72 | 0.79 | 4.24 | 1.07 | 3.19 | 0 | 4.24 | 3 |
| 2.68 | 1.63 | 0.94 | 4.34 | 0.73 | 0.18 | 12.08 | 3 |
| 3.14 | 1.62 | 1.23 | 5.41 | 0.46 | 0.12 | 13.35 | 3 |
| 2.75 | 2.63 | 1.44 | 4.96 | 0.58 | 0.3 | 15.08 | 3 |
| 0.72 | 0.79 | 4.24 | 1.07 | 3.19 | 0 | 4.24 | 3 |
| 0.59 | 0.43 | 39.62 | 39.72 | 0.79 | 0.13 | 2.86 | 1 |
| 0.19 | 0.15 | 31.95 | 31.66 | 0.57 | 0.06 | 0.95 | 1 |
| 0.09 | 0.19 | 20.18 | 18.66 | 0.4 | 0.09 | 0.78 | 1 |
| 0.12 | 0.16 | 19.67 | 17.73 | 0.3 | 0.11 | 0.78 | 1 |
| 0.2 | 0.16 | 36.2 | 32.71 | 0.7 | 0.27 | 1.01 | 1 |
| 0.1 | 0.12 | 21.52 | 15.29 | 0.06 | 0.09 | 0.62 | 1 |
| 0.99 | 1.03 | 11.66 | 10.75 | 2.37 | 2.26 | 5.69 | 1 |
| 4.17 | 2.67 | 1.06 | 6.38 | 0.76 | 0.6 | 18.73 | 2 |
| 6.52 | 2.83 | 1.25 | 6.76 | 0.89 | 2.95 | 26.22 | 2 |
| 3.77 | 2.02 | 2.18 | 5.81 | 1.08 | 1.08 | 16.64 | 2 |
| 3.11 | 2.83 | 1.65 | 6.54 | 0.71 | 0.34 | 16.65 | 2 |
| 1.72 | 1.84 | 4.92 | 6.87 | 0.97 | 0.64 | 9.98 | 2 |
| 4.45 | 2.12 | 0.98 | 5.44 | 0.65 | 0.34 | 18.42 | 2 |
| 4.39 | 2.08 | 1.4 | 6.62 | 0.57 | 0.68 | 18.14 | 2 |
| 1.41 | 0.32 | 4.64 | 0.15 | 3.55 | 0.87 | 4.85 | 3 |
| 1.47 | 1.14 | 2.62 | 5.46 | 3.45 | 0.8 | 5.45 | 3 |
| 1.75 | 2.53 | 2.12 | 4.1 | 1.29 | 0.89 | 12 | 3 |
| 4.2 | 2.16 | 1.42 | 6.75 | 0.83 | 0.2 | 17.83 | 3 |
| 9.01 | 5.09 | 0.96 | 4.41 | 0.94 | 9.61 | 39.54 | 3 |
| 1.47 | 1.14 | 2.62 | 5.53 | 9.39 | 0.8 | 5.45 | 3 |
| 1.05 | 1.05 | 8.04 | 2.84 | 5.12 | 1.98 | 5.88 | 3 |
