# Supplementary material for: Mine water inrush source discrimination model based on KPCA-ISSA-KELM
Source: PLoS One. 2024 Jun 3;19(6):e0299476. doi: 10.1371/journal.pone.0299476 (PMC11146743; doi:10.1371/journal.pone.0299476)
Supplement: S4 File — (DOCX) [file pone.0299476.s004.docx]

| **Dimension reduction data of a coal mine in Shanxi Province** | | | | | | |
| --- | --- | --- | --- | --- | --- | --- |
| **Y1** | **Y2** | **Y3** | **Y4** | **Y5** | **Y6** | **Type of water sample** |
| -0.0276 | -1.03013 | -0.41609 | 0.895261 | 0.176687 | -2.20948 | 1 |
| 0.376174 | 2.25193 | -0.08097 | 0.854638 | 0.011275 | -1.01696 | 1 |
| 0.235967 | 1.001629 | -0.20329 | 0.877356 | 0.113334 | -2.20085 | 1 |
| -0.23999 | -1.67251 | -0.41593 | 0.715058 | 0.075796 | -1.85123 | 1 |
| -1.17546 | -1.45324 | -1.00386 | 0.878055 | -0.05019 | -1.15952 | 1 |
| -0.58366 | -1.33158 | -0.75113 | 0.986351 | 0.058342 | -1.82165 | 1 |
| -0.24344 | -1.71583 | -0.37649 | 0.680955 | 0.106201 | -1.82954 | 1 |
| -0.07191 | -1.52001 | -0.47393 | 0.837086 | 0.097489 | -1.93196 | 1 |
| -1.82685 | -1.24442 | -0.2696 | 0.44772 | 0.114912 | -1.34654 | 1 |
| -0.18894 | -1.73453 | -0.36908 | 0.629797 | 0.135228 | -1.83822 | 1 |
| -0.48643 | -1.77015 | -0.55258 | 0.642054 | 0.031376 | -1.70626 | 1 |
| -0.06797 | 1.462778 | -0.45849 | 0.801573 | 0.097623 | -1.99253 | 1 |
| -0.1216 | -1.71697 | -0.37927 | 0.663823 | 0.142059 | -1.84411 | 1 |
| 1.821809 | -1.21576 | -0.26713 | 0.458881 | 0.144187 | -1.37228 | 1 |
| -0.10053 | -1.71307 | -0.3819 | 0.679408 | 0.152041 | -1.84232 | 1 |
| -2.01609 | -1.09228 | -0.23724 | 0.430647 | 0.076007 | -1.21757 | 1 |
| -0.71221 | -1.69217 | -0.71667 | 0.860255 | 0.080381 | -1.52047 | 1 |
| -0.18308 | -1.74443 | -0.37238 | 0.658488 | 0.118411 | -1.819 | 1 |
| -0.96151 | -1.58714 | -0.36278 | 0.642242 | 0.122053 | -1.72483 | 1 |
| 1.474395 | -0.98535 | -0.49853 | 0.775523 | 0.489583 | -0.07571 | 1 |
| -0.25587 | -1.7202 | -0.38192 | 0.668316 | 0.131348 | -1.82607 | 1 |
| 0.127288 | 0.549456 | -0.4745 | 1.326939 | 0.042063 | -2.16418 | 1 |
| -0.12229 | -1.60197 | -0.39998 | 0.739269 | 0.047596 | -1.91727 | 1 |
| -0.002 | -1.40165 | -0.3851 | 0.719754 | 0.195086 | -2.07448 | 1 |
| 0.008032 | -1.54834 | -0.36572 | 0.562062 | 0.3436 | -2.00244 | 2 |
| 0.598688 | 2.07361 | 0.20651 | 0.722012 | 0.163228 | -1.31001 | 2 |
| 0.601084 | 1.907856 | 0.149133 | 0.778882 | 0.177273 | -1.51789 | 2 |
| 0.464695 | 2.126256 | 0.185165 | 0.700355 | 0.151462 | -1.3097 | 2 |
| 0.65426 | 2.019143 | 0.271971 | 0.722695 | 0.145526 | -1.3623 | 2 |
| -0.00247 | -0.26326 | 2.3453 | 1.005085 | -0.40291 | 0.08872 | 2 |
| 0.189547 | 2.162444 | -0.14551 | 0.977849 | 0.108566 | -1.13769 | 2 |
| 0.604884 | 2.011287 | 0.199358 | 0.758805 | 0.167815 | -1.38552 | 2 |
| -0.14473 | 2.295628 | 0.46587 | 0.388984 | -0.50439 | -1.03957 | 2 |
| 0.371666 | 2.211988 | -0.01713 | 0.882846 | 0.061261 | -1.08881 | 2 |
| 0.618707 | 2.070418 | 0.231823 | 0.748424 | 0.090277 | -1.3042 | 2 |
| 0.643103 | 2.044427 | 0.235305 | 0.713718 | 0.147147 | -1.3314 | 2 |
| 0.616326 | 2.051239 | 0.22258 | 0.69898 | 0.119502 | -1.35048 | 2 |
| 0.590151 | 1.995571 | 0.188608 | 0.715148 | 0.121419 | -1.45095 | 2 |
| -0.03287 | 2.378311 | 0.220253 | 0.557032 | 0.151672 | -0.9581 | 2 |
| 0.547435 | 1.968146 | -0.11136 | 0.820976 | 0.174593 | -1.40304 | 2 |
| -0.50692 | 2.04343 | 0.040031 | 0.371223 | -0.48183 | -1.44151 | 2 |
| 0.157157 | 1.946411 | -0.09187 | -0.25432 | 1.033445 | -1.42969 | 2 |
| 0.6349 | 1.786108 | 0.165042 | 0.802249 | 0.233295 | -1.62798 | 2 |
| 0.556018 | 2.054125 | 0.109369 | 0.765937 | 0.119107 | -1.34941 | 2 |
| 0.415777 | 1.178961 | -0.23217 | 0.839918 | 0.291552 | -2.10866 | 2 |
| 0.630311 | 2.003471 | 0.149288 | 0.734075 | 0.181955 | -1.39024 | 2 |
| 0.604762 | 1.749037 | -0.03886 | 0.809833 | 0.274212 | -1.64929 | 2 |
| -0.01454 | -1.58515 | -0.35137 | 0.545879 | 0.331306 | -1.98489 | 2 |
| 0.488426 | 1.881833 | -0.11715 | 0.853756 | 0.19696 | -1.53494 | 2 |
| 0.630277 | 1.649789 | 0.107216 | 0.800003 | 0.239175 | -1.7615 | 2 |
| 0.531004 | 2.061029 | 0.022333 | 0.72917 | 0.16223 | -1.35722 | 2 |
| -0.04981 | -1.39254 | -0.41601 | 0.592651 | 0.325012 | -2.09263 | 2 |
| -0.2781 | -1.74017 | -0.3691 | 0.490373 | 0.251364 | -1.85554 | 2 |
| 0.44633 | 1.232337 | -0.18578 | 0.802687 | 0.285589 | -2.08904 | 2 |
| -0.12357 | -1.66521 | -0.35658 | 0.524504 | 0.288421 | -1.92443 | 2 |
| 0.522395 | 1.757617 | -0.0423 | 0.83958 | 0.194961 | -1.67718 | 2 |
| 0.413049 | 1.002975 | -0.25828 | 0.842702 | 0.340544 | -2.18636 | 2 |
| 0.615835 | 1.805622 | 0.114717 | 0.804965 | 0.257443 | -1.61169 | 2 |
| 0.106626 | 2.166649 | -0.33772 | 1.058267 | 0.113049 | -1.01302 | 2 |
| -0.09027 | 0.972717 | -0.86856 | 1.328932 | 0.326648 | -1.84884 | 2 |
| 0.527065 | 2.078441 | 0.21272 | 0.703736 | 0.172659 | -1.33675 | 2 |
| -0.22897 | 0.265446 | -0.67397 | 1.224933 | 0.310038 | -2.19661 | 2 |
| 0.527064 | 1.947399 | 0.098267 | 0.742782 | 0.219825 | -1.49889 | 2 |
| 0.646867 | 1.967312 | 0.160357 | 0.730663 | 0.190539 | -1.43363 | 2 |
| 0.184172 | 2.173286 | -0.38635 | 1.023059 | 0.119421 | -1.01588 | 2 |
| 0.634697 | 2.025701 | 0.163817 | 0.731504 | 0.176814 | -1.36071 | 2 |
| 0.627369 | 1.964034 | 0.162232 | 0.774457 | 0.189465 | -1.43275 | 2 |
| 0.11413 | 2.257368 | -0.25636 | 1.011206 | 0.071306 | -0.86906 | 2 |
| 0.578968 | 2.056542 | 0.227713 | 0.762588 | 0.09202 | -1.3362 | 2 |
| 0.41262 | 1.896025 | 0.315937 | 0.81027 | 0.115906 | -1.56978 | 2 |
| 0.646765 | 1.967003 | 0.160466 | 0.731608 | 0.191725 | -1.43349 | 2 |
| 0.638 | 2.003733 | 0.225744 | 0.706774 | 0.15961 | -1.39357 | 2 |
| 0.823581 | 1.983777 | 0.09779 | 0.682863 | 0.206825 | -1.34349 | 2 |
| 0.542135 | 1.824508 | -0.03246 | 0.777374 | 0.230296 | -1.61964 | 2 |
| -0.20928 | -1.72292 | -0.36701 | 0.525781 | 0.24158 | -1.87152 | 2 |
| -0.83084 | 0.549293 | 0.013568 | -1.37075 | 1.277432 | -1.57811 | 3 |
| -0.2954 | 1.528909 | -0.13791 | -0.92571 | 1.333103 | -1.32394 | 3 |
| -0.18127 | 1.831929 | 0.243376 | -0.5995 | 0.678963 | -1.64304 | 3 |
| -1.31876 | 0.762575 | -0.12395 | -1.07386 | 0.010692 | -1.83325 | 3 |
| 0.62597 | 2.009571 | 0.188093 | 0.443958 | 0.446969 | -1.44495 | 3 |
| 0.684843 | 1.900513 | 0.21461 | 0.559775 | 0.437099 | -1.52553 | 3 |
| 0.520831 | 2.032403 | 0.049175 | 0.570383 | 0.432473 | -1.40084 | 3 |
| -1.31876 | 0.762575 | -0.12395 | -1.07386 | 0.010692 | -1.83325 | 3 |
| 0.003943 | -1.67262 | -0.37759 | 0.724645 | 0.100843 | -1.86772 | 1 |
| -0.01414 | -1.73415 | -0.37712 | 0.688786 | 0.111244 | -1.82541 | 1 |
| -0.12202 | -1.72482 | -0.38289 | 0.668531 | 0.140074 | -1.83457 | 1 |
| -0.16162 | -1.72078 | -0.37929 | 0.673283 | 0.148711 | -1.83422 | 1 |
| -0.15695 | -1.73375 | -0.38443 | 0.691253 | 0.103241 | -1.81773 | 1 |
| -0.51603 | -1.69805 | -0.36742 | 0.669646 | 0.165291 | -1.79263 | 1 |
| -0.14498 | -1.01102 | -0.662 | 0.834306 | -0.11078 | -2.19101 | 1 |
| 0.621145 | 2.026527 | 0.117934 | 0.739019 | 0.186115 | -1.35638 | 2 |
| 0.464042 | 2.163098 | 0.093605 | 0.833799 | 0.086504 | -1.18044 | 2 |
| 0.491967 | 1.981927 | 0.066292 | 0.750377 | 0.152558 | -1.48622 | 2 |
| 0.643744 | 1.911443 | 0.010479 | 0.738203 | 0.251045 | -1.47595 | 2 |
| 0.413049 | 1.002975 | -0.25828 | 0.842702 | 0.340544 | -2.18636 | 2 |
| 0.526912 | 2.07784 | 0.212915 | 0.705562 | 0.174937 | -1.33653 | 2 |
| 0.627369 | 1.964034 | 0.162232 | 0.774457 | 0.189465 | -1.43275 | 2 |
| -1.667 | 1.053545 | -0.01644 | -0.89959 | -0.24814 | -1.48816 | 3 |
| 1.107993 | 0.637624 | -0.21582 | -0.4655 | -0.10068 | -2.24087 | 3 |
| 0.389488 | 2.021706 | -0.17816 | 0.448662 | 0.449807 | -1.47739 | 3 |
| 0.654675 | 1.952461 | 0.198079 | 0.626014 | 0.290725 | -1.48027 | 3 |
| 0.191584 | 2.365236 | -0.11878 | 0.858574 | 0.096428 | -0.75798 | 3 |
| 0.700644 | 0.382121 | -0.29066 | -1.51233 | -1.47086 | -1.29534 | 3 |
| -1.47018 | 0.145294 | -0.68676 | -0.45839 | -0.51564 | -1.96684 | 3 |
